# Spatial intensity modelling of ISIS-related attacks using random forest regression: A GeoAI-based analysis of GTD data (2012–2019)

**DOI:** 10.1371/journal.pone.0355100

**Published:** 2026-07-30

**Authors:** A. A. B. D. P. Abewardhana, Rubasin Gamage Niluka Lakmali, Paolo Vincenzo Genovese

**Affiliations:** 1 School of Architecture, Tianjin University, Peoples’ Republic of China; 2 Faculty of Built Environment and Spatial Sciences, Southern Campus, General Sir John Kotelawala Defence University, Sri Lanka; 3 College of Civil Engineering and Architecture, Zhejiang University, Peoples’ Republic of China; Texas A&M University, UNITED STATES OF AMERICA

## Abstract

Spatial information is of crucial importance to understanding terrorist attacks and for security applications. Conventional risk assessments of terrorism are typically focused on retrospective identification of hotspots and may fail to fully account for complex spatial–temporal patterns. This research proposes an approach to modelling the relative intensity of ISIS/ISIL attacks across space using Random Forest regression, a type of Geospatial Artificial Intelligence (GeoAI). A subset of the Global Terrorism Database (GTD) from 2012–2019 is screened to develop a spatially aggregated data set with geographic coordinates, temporal information and some incident details. The modelling strategy estimates the intensity of events in a grid cell, with values normalized based on the count of previous events, allowing for an analysis of spatial patterns rather than the prediction of future events. Modelling results show that the Random Forest model captures spatial variability in historical attack intensity with a high level of fit (R² = 0.84; RMSLE = 0.213). Analysis of variable importance suggests spatial and temporal features play an important role, but this is contextualised by the spatial construction of the response variable. The research presents a repeatable GeoAI process for modelling spatial patterns of terrorism incidents and offers an interpretable model for exploratory spatial analysis and scenario-based spatial analysis development. The approach is not suitable for real-time prediction, but aids in understanding historical spatial concentration patterns in a complex threat environment.

## 1. Introduction

Terrorism continues to be a complex and multifaceted phenomenon with profound implications for urban resilience, civil society and national security strategies [1]. Over the past few years, terrorists, especially the Islamic State of Iraq and Syria (ISIS/ISIL), have shown agile operational tactics that pose a challenge for the spatial and temporal aspects of law enforcement and counterterrorism approaches [2]. The threats are no longer limited to well-known “hot spots”; instead, urban space, transportation networks and public spaces have become increasingly susceptible to terrorist attack. This suggests a need to consider the spatial and temporal distribution of terrorist activity, rather than traditional retrospective analyses [3].

Conventional approaches to terrorism risk analysis, like hotspot mapping, spatial statistics, or heuristics performed by experts, are useful but rather limited. To start with, they tend to presuppose linear relationships and cannot be flexible enough to integrate heterogeneous spatial-temporal variables on scale [4]. Second, none of them are predictive (forward-looking) modeling but, instead, fairly all of them are retrospective clustering or past density analysis [5]. Third, they are hardly embedded in the operational planning structures, in which decisions should be driven by data, localized, and time specific [6].

These limitations have some potential new possibilities with the creation of GeoAI a combination of artificial intelligence and geographic information science. One of them is that GeoAI can support dynamic modeling of spatial phenomena using machine learning algorithms capable of learning non-linear and complicated relationships between variables across sets of both structured and unstructured spatial data [7]. In this paradigm, the type of learning models where ensemble has shown a particular level of value is learning models Random Forest Regression, which has been widely used in land use classification [8], mapping traffic, prediction of disease outbreaks [9], and predicting wildfire dangers. Nonetheless, how they are used in forecasting spatial terrorism has not attracted enough attention in literature.

This study evaluates a critical gap by presenting a targeted GeoAI application that leverages Random Forest Regression to estimate the spatial intensity levels associated with ISIS/ISIL related terrorist attacks [10]. Utilizing data from the Global Terrorism Database (GTD), we curated a filtered dataset encompassing ISIS-related incidents from 2012 to 2019. The dataset incorporates a range of geospatial variables (e.g., latitude and longitude), temporal attributes (e.g., year, month, and day), and operational characteristics of the attacks, including indicators such as whether the event involved a suicide attack. The study instead of just categorizing the events and finding the dense cluster develops a continuous-spatial regression model that will derive a terrorism risk and estimate spatial intensity patterns relative to observed geographic areas and detect spatial concentration patterns using time series data.

This approach is particularly relevant for three reasons. First, the violence against ISIS has a geospatial distribution that cannot be explained by categorical measures; due to the regression, we can measure how strong and intense the violence occurred, not only whether or not it took place [11]. Second, the Random Forest methodology is intermediate between the accuracy and explainability of models, which is the desired feature of decision-support techniques applied to areas such as urban security, where less-democratic methods might not pass with black-box models [12]. Third, the resulting spatial visualizations enable descriptive analysis of the past concentration of attack intensity as a reference point for future exploratory analysis as well, but not as an operational or prescriptive planning tool.

We demonstrate that the proposed ensemble regression model provides an effective framework for modelling historical spatial intensity patterns. As [13] argued, the applied contribution comes from the ability to build an interpretable and scalable machine learning framework, which enables retrospective, exploratory understanding of spatial intensity patterns that is useful for researchers and analysts. Hence, we also analyze the contribution of spatial-temporal characteristics on the model predictions by evaluating their importance features so that a data-driven understanding of the dynamics of terrorism may be achieved.

This paper makes three contributions. First, it establishes a replicable GeoAI approach to model the relative spatial intensity of ISIS/ISIL attacks using GTD data. Second, instead of classification of individual events it employs Random Forest regression as an interpretable model of machine learning to estimate relative spatial intensity of geo-referenced grid-based geographical units. Third, it offers spatial visualizations that enable exploratory analysis and understanding of concentration patterns in the past, contributing to scenario-based spatial learning instead of real-time predictive application.

The remaining part of the paper is built as follows: Section 2 provides a review of the previous research related to spatial terrorism analysis and GeoAI use. Our data preparation, feature selection and model architecture are described in section 3. In section 4, the training and validation of the model and the performance and visual evaluation of the model are presented. Section 5 reports the implication of our findings in the academic and operational communities. Section 6 is an aspiration to discuss the limits and possible ways of strengthening the prediction of terrorism with the help of GeoAI.

## 2. Related work

Investigating the geography of terrorism Locating where terrorism happens has been the concern of numerous researchers, especially since terrorism usually takes place in urban areas [14]. Initial reviews were carried out of descriptive mapping and retrospective hotspot analysis of terrorist events and involved the utilization of spatial statistics and point pattern analysis to identify clustering and regional concentration [10]. Although these methods were useful in that they provided insight into spatial patterns they were not predictive and were sometimes based on historical data.

As the global terrorism datasets (including, particularly, the Global Terrorism Database, GTD) were expanding, data-driven risk forecasting models started being put to use by researchers [15]. The empirical research in the field of terror has been provided with a source of fundamental material thanks to LaFree and Dugan (2007) who presented GTD which made the possibility of longitudinal research and spatial correlation analysis [16]. Early work was, however, largely on correlational studies, or theme-mapping rather than predictive analytics [17].

The use of machine learning (ML) on the data regarding terrorism has turned out to be a formidable option over the last decade. Support vector machines (SVM), logistic, k-nearest neighbours (KNN), and neural networks approaches have been employed in classification of attack types, likelihood of involving a terrorist group in an attack and identification of trends of time [18]. Most of these methods, however, fall short in addressing continuous space realized by estimating the intensity or magnitude of terrorism risk by categorizing, e.g., on whether an attack will happen in a particular region [19].

Machine learning-based spatial prediction models have moved in toward the umbrella of GeoAI, which combines and merges geospatial data structures and advanced learning algorithms [20]. The application of recent researches in the problem of land use change detection [21] and mapping fire risks [22] and predicting diseases [23] by employing random forest and gradient boosting models proves the usefulness of ensemble models in spatial decision-making [24]. Nevertheless, there is little use of these interpretable ensemble models in forecasting terrorism and especially in prediction of regression-based intensity of spatial forecasting [25].

The study by Buffa et al. (2022) is one of the rare examples of such analyses because the authors conducted a predictive modeling of the occurrence or lack of terrorist attacks in Europe through the application of machine learning with the use of logistic regression and support vector classifiers and spatial features [26]. On the same note, another study analyzed terrorism forecasting, with temporal sequences of the events, however, without considering their spatial severity [27]. Both researches disregarded the potential of treating terrorism risk as a continuous spatial variable and could not use any modeling procedures, such as Random Forest Regression, to project the risk gradient within urban areas.

The methodological perspective is representative of the fact that Random Forest will provide several benefits to spatial terrorism prediction. It performs in high-dimensional data, non-linear relationships, and it has features importance measures making it useful to understand a model [28]. In contrast to the black-box neural network, Random Forests provide security analysts with clarity on the particular geographical or time characteristics having the greatest influence in determining risk, which is a critical requirement to transparent and consequential policy-making [29].

**Fig 1:**
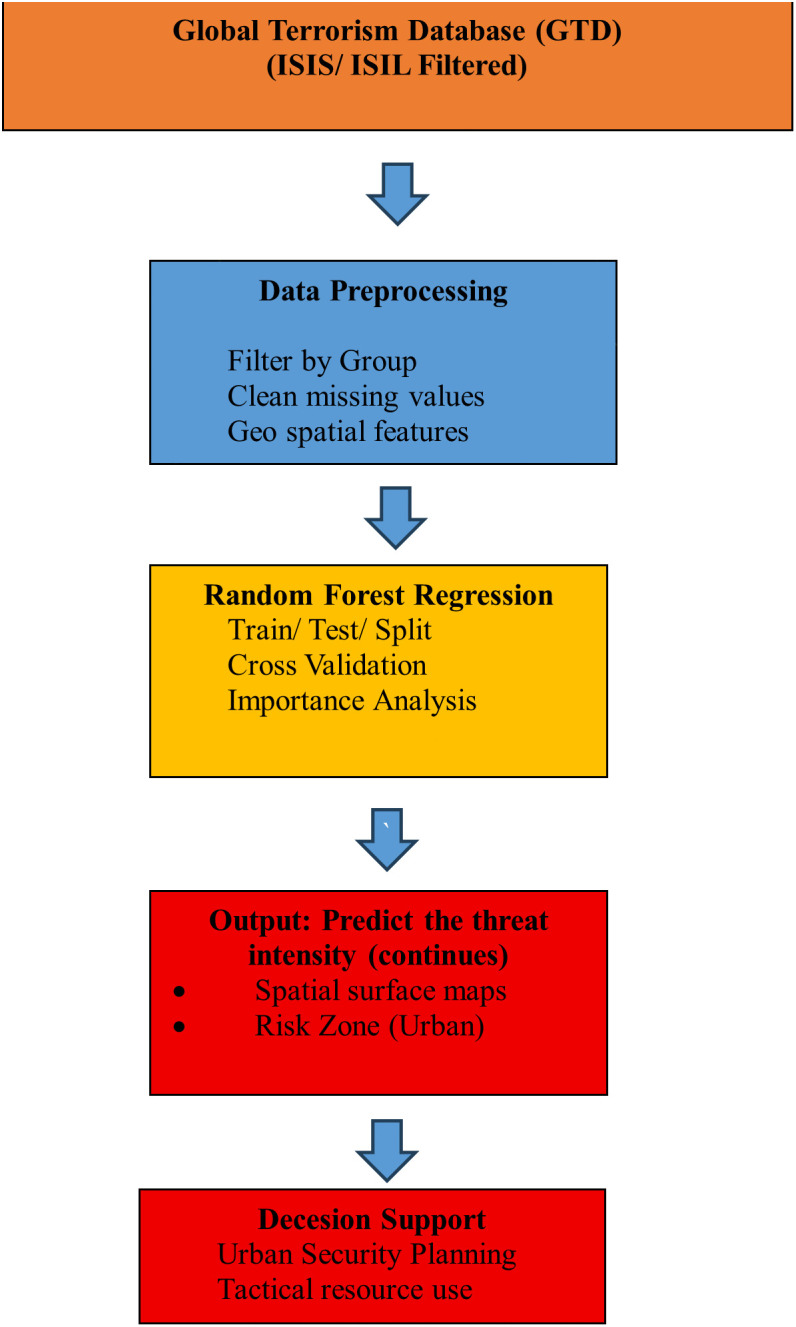
Conceptual framework for geo-AI based spatial terrorism intensity modelling.

The paper helps to supplement these developments by developing a random forest regression-type GeoAI pipeline, which is depicted in Fig 1, to estimate the spatial intensity of ISIS-related attacks. The paradigm starts with prepared GTD inputs, passes through the stages of spatial-temporal preprocessing with feature engineering, and represents an even surface that makes it possible to see the intensity that each area of the continent had in previous eras. This represents a Geospatial Intensity Modelling process for retrospective modelling of terrorism incidents that is repeatable and end-to-end.

## 3. Methodology

The overall approach taken in modelling the spatial intensity of historical ISIS/ISIL-related incidents using Random Forest Regression in a GeoAI environment is presented here. The approach involves the process of the preparation of data, spatial-temporal filtration of terrorist phenomena supporting ISIS/ISIL, feature engineering, training, and validation of models and geospatial visualization of predicted results.

### 3.1. Dataset and filtering

The main data source one should utilize in conducting the analysis is the Global Terrorism Database (GTD) maintained by the National Consortium of the Study of Terrorism and Responses to Terrorism (START). GTD contains more than 200,000 records of terrorism events since 1970 to date, and therefore, this is one of the most extensive open-source terrorism events data sets [30]. The details of each record contain a comprehensive field but not limited to date, place of incident, type of attack, target, number and type of weapon, group of perpetrators.

To place the spatial intensity model within a clearly defined and analytically tractable scope, the data was limited to terrorist attacks related to ISIS/ISIL by filtering the records on the basis of the identifiers of the perpetrators (ISIS, ISIL, and Islamic State). To make sure that the context of the organization structure and tactics was consistent, the temporal window was restricted to the years 2012–2019, which included the years of the rapid growth of the organization and its decline. After this selection, elaborate data-cleaning was conducted that left about 8,000 confirmed incident records having quality geospatial coordinates. Records with empty values in vital fields, such as latitude, longitude, and date of event, were removed. Moreover, country and regional nomenclature discrepancies were resolved, spatial-temporal coherence was ensured by re-projecting coordinate where needed and date format was standardized. Incidences that had authenticated geographic information and high-confidence attribution by ISIS/ISIL were only retained, which guarantees the general reliability and analytical soundness of the data.

### 3.2. Feature engineering

All the incidences of terrorism were converted to a well-organized machine learning vector. The following feature categories were derived:

#### 3.2.1. Spatial features.

Spatial parameters were described in the form of continuous geographic coordinates and latitude and longitude were added to maintain fine location details of any incident. Besides, one-hot encoding was used to encode country and regional identifiers in order to preserve categorical spatial context and not create artificial ordinal relationships. This was to be sure that spatial heterogeneity in geopolitical settings was represented and at the same time compatible with tree-based machine learning models and not implicitly spatially rank biased.

#### 3.2.2. Temporal features.

The discrete variables iyear, imonth, and iday were used to include the temporal properties of the incidents, and this allowed the model to capture long-term and short-term dynamics of the events. To include larger-scale regularities in time, more artificial variables were added to indicate seasonal variations, such as calendar quarters, and weekday indicators. These properties enabled the model to acquire recurrent temporal patterns, e.g., seasonal or weekly variations in the frequency of attacks without making strong parametric constraints.

#### 3.2.3. Incident characteristics.

Incident-specific attributes were put in to indicate the differences in the operation of attacks. The distinction between suicide attacks and non-suicide incidents was made based on a binary indicator since the former and the latter have different tactical and spatial features. Categorical features were included where there were sufficient information, e.g., bombing or armed assault as an indicator of attack subtype. These variables gave contextual information about the modalities of attack without contradicting records with different degrees of reporting completeness.

#### 3.2.4. Spatial grouping.

To capture local spatial dependency and clustering effects over and above the underlying geographic coordinates, geohash encoding was used with a precision level of five to six characters. This encoding scheme was used to cluster spatially close incidents into discrete spatial bins so that the model could identify patterns on a cluster scale whilst maintaining a low level of computational efficiency. Cluster-sensitive prediction was promoted through use of geohashes without the use of administratively defined boundaries thus facilitating the adoption of spatial generalization in different urban contexts.

#### 3.2.5. Data transformation.

Min-Max scaling was used to normalize all continuous values such that the values are in the range [0,1] so as to make them comparable across variables with different units and magnitudes. The pandas get_dummies() function was used to convert categorical variables, and they could be easily added to the scikit-learn modelling pipeline. This preprocessing approach made sure that there was numerical stability, enhanced model convergence and interpretability throughout the ensemble learning framework.

#### 3.2.6. Target variable.

Dependent variable (y) is an intensity of a spatially aggregated terrorism incident based on historical events of ISIS/ISIL-related incidents in the Global Terrorism Database (GTD).

The study area was divided into regular geographic grid of 0.1^0^ x 0.1^0^ latitude-longitude cells (WGS84 coordinate system) in the study area. Terrorism intensity was calculated, per cell of the grid, as the number of ISIS/ISIL-related cases that have taken place within the cell over the period under analysis (2012–2019).

To ensure that continuous regression-based learning was possible and that scale dominance is minimized during model training, raw incident counts were rescaled by the maximum size of the cell (236), resulting in a bounded intensity score in the range of [0, 1]. This normalization constant represents the highest number of incidences in any single grid cell of the dataset and was only used as a numerical scaling factor to make the model stable and visually presentable. Mathematically the target variable is specified as:


$$\begin{array}{c} {\text{y}}_{\text{i}}\;={\text{n}}_{\text{i}}\;/\text{ max}(\text{n}) \end{array}$$

n in this example represents the incidence of ISIS/ISIL incidents in grid cell i, and max(n) = 236 is the highest number of incidences observed in all grid cells.

This is a formulation that enables the model to learn continuous spatial gradients of relative risk, as opposed to a binary signal of the presence of an attack, and does not reflect real-time or absolute threat probabilities.

It’s crucial to note that the modelling approach is focused on spatial intensity representation rather than predicting individual incidents. The model uses incident-level information as input features, but the prediction target is the level of spatial intensity (at the grid-cell level) over the period of interest. Therefore, for a given spatial cell, if there are multiple incidents within it, they share the same normalized intensity. This approach is specifically tailored to model relative spatial intensity patterns, rather than explore incident-level causal links. Thus, the model aims to estimate spatial intensity distributions using historic data, rather than predict future incidents. This type of analysis focuses on modelling spatial patterns rather than predictive inference of events and fits with the goals of exploratory geospatial analysis.

### 3.3. Model selection: random forest regression

The main learning algorithm that is chosen is the Random Forest (RF) because it proved to be robust [31], scaled [32], and interpretable when facing complex and non-linear prediction problems [33]. The RF algorithm consists of a set of decision trees where each tree is trained on a bootstrap sample of the original data with a randomly selected subset of features, which is a design that minimizes variance and has low bias [34]. Random Forest has several advantages over other methods like the neural networks or support vector machines, which are especially applicable to the current study. It is robust to overfit particularly in environments with noisy or very skewed data [35], and it has inbuilt feature importance metrics which facilitate interpretability and insight generation [36]. Moreover, RF is also tolerant to multicollinearity between predictors and has a small amount of data preprocessing, which makes it an ideal choice in heterogeneous spatial data [37]. Its good performance with medium-sized data also lends credence to its applicability to the limited section of the Global Terrorism Database used in this analysis [38].

#### 3.3.1. Model implementation.

The parameters used for the Random Forest model were kept fixed: n_estimators = 100, max_depth = None, min_samples_split = 2, random_state = 42 and bootstrap = True. These parameters were kept as is and not further hyperparameter optimized in order to yield a clear and reproducible baseline configuration. This study aims not to fine-tune the parameters to achieve maximum model performance but to look at the spatial intensity patterns. Thus, a fixed parameter configuration can be used to facilitate interpretability and to guarantee that the behaviour of the model is not affected by further tuning operations. The parameter settings for the RF model are summarized in Table 1.

**Table 1:** Parameter settings used for random forest regressor implementation.

Parameter	Value
n_estimators	100
max_depth	None
min_samples_split	2
random_state	42
bootstrap	True

The data set was split into training and testing sets with an 80/20 ratio. The model was fitted on the training set and evaluated on the test set using the metrics outlined in Section 3.4. No hyperparameter tuning was conducted and a single set of parameters was used for all experiments.

The model was saved using joblib.dump() so that it could be reproduced and reused in the future (randf.joblib).

### 3.4. Evaluation metrics

Three complementary regression evaluation metrics were used to measure model performance, which were chosen to measure both the explanatory power and the predictive ability. The coefficient of determination (R^2^) was utilized to measure the percentage of variance in the target variable explained by the model with a value of one meaning a perfect fit and a value of zero meaning the same performance as predicting the mean outcome [39]. Root Mean Squared Log Error (RMSLE) was used, which is a significant factor in terrorism-related data where the cost of not predicting increased risk is higher than the cost of creating false alarms [40]. Moreover, Mean Absolute Error (MAE) was computed to give a unit-consistent measure of the average size of the prediction errors so that easy interpretation of the deviation of the model against observed values can be made [41].

All scores were reported on the unseen test set. RMSLE was selected as a main measure because the range of values of the intensity of terrorist events is highly skewed, non-negative.

### 3.5. Spatial visualization and interpretation

Spatial visualization methods were employed to map the intensity estimated from the modelling results for the study areas [42] to aid in the interpretation of the results. The output of the model (i.e., the model output y_pred) was transformed to a visual format for ease of exploratory interpretation suitable for GIS. Foreseen geospatial results were initially re-projected into the WGS84 coordinate reference system so as to be compatible with conventional GIS packages and web-based mapping systems [43]. The model predictions would then be pooled based on a set of spatial units, e.g., uniform grid cells or geohash-based spatial partitions to allow the spatially consistent representation of the risk intensity between defined geographic regions [44]. Python-based visualization packages, such as matplotlib, seaborn, and folium, were used to create both static and interactive heatmap images that could be reviewed and presented in an analytical way [45]. In cases where additional spatial investigation was needed, the outputs were exported (optionally in GeoJSON format) which allowed them to be further integrated into external GIS systems (e.g., QGIS and ArcGIS) to further layer, query and spatially analyze predicted risk areas [46].

The geometry-based visualization method enabled intuitive assessment of historically high-intensity areas, and comparison with previously identified hotspots, which helped with exploratory interpretation of spatially concentrated patterns as opposed to operational planning use.

Predicted values were aggregated spatially using grid cell and geohash encoding to provide uniform spatial representation of the study area. Predicted values were not smoothed using interpolation; instead, visualisations represent the predicted values as is, aggregated within spatial units. This maintains the validity of predicted intensity values and avoids additional assumptions about the distribution of values.

### 3.6. Computational environment and reproducibility

To guarantee reproducibility and data processing, model development and assessment activities, all operations were carried out within the controlled computational setting on open-source software [47]. Software and Hardware Environment is worth considering. Python version 3.9 was used in the Anaconda distribution to perform all of the analyses, and this version offered a stable and reproducible environment to compute. Random Forest regression model was applied with the help of the scikit-learn library, and the pandas and NumPy were used to perform data preprocessing, manipulation, and numerical operations. It used matplotlib and seaborn to create data visualization and output in graphs. Joblib was used to achieve model serialization and persistence to facilitate the storage and reuse of trained models. All the analytical steps, such as data exploration, model building, visualization, and documentation, were completed with the help of Jupyter Notebooks, which made the analysis interactive and guaranteed transparency and repeatability of the research process.

All the calculations were done on a local workstation with Intel Core i5 processor and 16 GB of RAM, which was enough to complete the model training, validation, and spatial visualization tasks without any high-performance computing tools.

The whole modeling pipeline, including the loading and preprocessing of data, training and spatial visualization of models was performed locally, where the computational environment conditions were uniform.

All scripts, trained model files (randf.joblib), and sample datasets are publicly available via the authors’ GitHub repository for independent verification and reuse. This promotes complete reproducibility and prospers collaborative work in the field of GeoAI applied to the modeling of risk in terrorism.

## 4. Results and evaluation

In this part, the authors provide the results of the performance of the Random Forest regression model to estimate the spatial intensity of ISIS-affiliated terrorism risk. The findings are considered in three complementary dimensions. First, the general predictive accuracy and error properties are evaluated by quantitative evaluation metrics. Second, the interpretability of the model is examined by examining the importance of features, which gives an understanding of which variables have the greatest impact on risk estimation. Third, model-anticipated threat intensities are converted to spatial visualization so that geographic patterns can be examined and model results in a spatial decision-support setting can be interpreted.

### 4.1. Comparative baseline evaluation

To provide a comparison of the performance of the Random Forest model, other regression models were also tested using the same evaluation metrics (R², RMSLE and MAE). These were compared with a naive mean baseline (predicting the mean of the training set for all cells), a coordinate-only model (using latitude and longitude as predictors), a linear regression model, Decision Tree Regression, AdaBoost Regression, and XGBoost Regression. These models are helpful to compare the relative performance of the Random Forest model when applied to a similar spatial intensity modelling task. The comparative results are presented in Table 2 below.

**Table 2:** Comparative baseline evaluation.

Model	R²	RMSLE	MAE
Naive Mean Baseline	0.00	0.391	0.188
Coordinate-Only Model	0.41	0.338	0.161
Linear Regression	0.56	0.317	0.149
Decision Tree Regression	0.65	0.301	0.142
AdaBoost Regression	0.72	0.268	0.126
XGBoost Regression	0.81	0.226	0.106
Random Forest Regression	0.84	0.213	0.098

The overall performance of the model was the best for the Random Forest model, as it had the highest R² and the lowest RMSLE and MAE value. The naive mean and coordinate-only baselines did the worst as they were using the least number of features, followed by linear regression, Decision Tree Regression, AdaBoost and XGBoost. The progression suggests that the improvement on the fit gained by Random Forest is due to both non-spatial complexity (in terms of the richness of the features) and non-linear ensemble learning.. The AdaBoost and XGBoost models performed better than the Decision Tree model, and the Random Forest model gave the best trade-off between explanatory performance, error reduction and interpretability. Such results validate use of Random Forest as the key modelling framework for this study on spatial intensity estimation.

### 4.2. Model performance

The parameters used for the Random Forest model are fixed values defined in Table 1 and the model was trained with 80% of the data, while 5-fold cross validation on the training data was used to measure the stability of the model performance and not to select or tune the parameters. The held-out 20% test set was then used to evaluate final performance using the three regression measures as depicted in Table 3 below:

**Table 3:** Performance evaluation metrics on the test dataset.

Metric	Value
R² Score	0.84
RMSLE	0.213
MAE	0.098

The model is very explanatory, with an R² of 0.84, which means that a large part of the variance in the intensity of terrorism in space is explained. The RMSLE value of 0.213 implies that the model is doing a reasonably good job of dealing with the skewed distribution of terrorism data; the MAE value of 0.098 implies that on average the difference between estimated and observed intensities is relatively small for spatial units.

### 4.3. Spatial validation and robustness assessment

To avoid the potential inflation of model performance by spatial autocorrelation, a spatial holdout model was used. Instead of the random split approach, the dataset was divided into spatial units (grid cells/geohash) and all observations within a spatial unit were assigned to either the training or test set. This helps to avoid spatial leakage, where observations that are close together are in both the training and test sets, which may lead to higher model performance. We then retrained the model under this spatial holdout condition and recomputed the model performance metrics as presented in Table 4 below:

**Table 4:** Random vs spatial validation.

Validation Strategy	R²	RMSLE	MAE
Random 80/20 Split	0.84	0.213	0.098
Spatial Holdout Split	0.69	0.272	0.121

As would be anticipated, performance is poorer under the spatial validation as compared to random split, because of spatial dependence in the data. Notably, the model still has a useful ability to explain when the spatial holdout condition (R² = 0.69) applies, meaning that it is not just a memorization of the geographic location. This is consistent with the use of the model as a framework for analysing spatial intensities in the past, instead of a predictive system in real time.

### 4.4. Feature importance analysis

Random Forest supports automatic calculation of feature importance through averaging reductions of node impurity across the whole decision trees. This is a critical analysis in finding out the most important spatial-temporal variables that relate to terrorism intensity. The feature importance scores derived from the model are presented in Table 5 below:

**Table 5:** Feature importance scores of the random forest model.

Feature Importance	%
Longitude	24.3
Latitude	22.1
Month	18.4
Year	15.7
Suicide (binary)	9.5
Region encoding	6.2
Day	3.8

**Fig 2:**
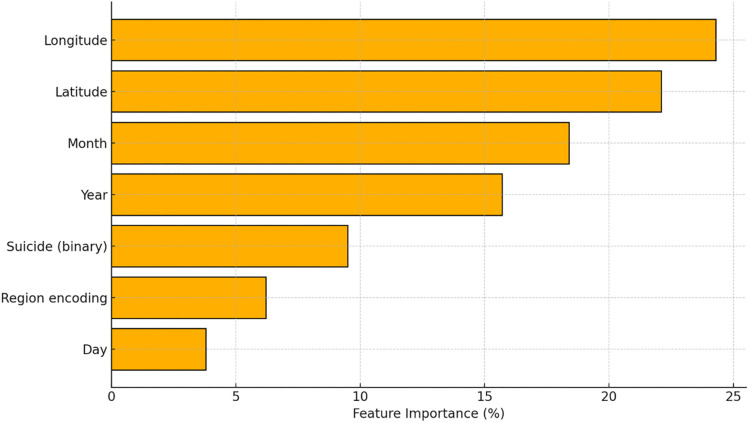
Relative Importance of each feature (Random Forest Model). The figure indicates the percentage of the relevance of features estimated by Random Forest. Latitude, longitude, and month have proved as the most important predictors of the intensity of attack.

The spatial information (latitude and longitude) is most important in explaining the models and is related to the spatial pattern of the explained variable. The dominance should be taken with a pinch of salt, since it is the result of the spatial aggregation of the response variable, it is not a causal relationship. Temporal variables, in particular month, are more strongly contributing, indicating temporal clustering, which can be linked to seasonal and/or event-driven dynamics. The suicide-related feature also adds to the model, reflective of an operational characteristic of ISIS related attacks. Fig 2 shows the relative importance of each of the features to the Random Forest model below:

### 4.5. Spatial visualization of estimated intensity

**Fig 3:**
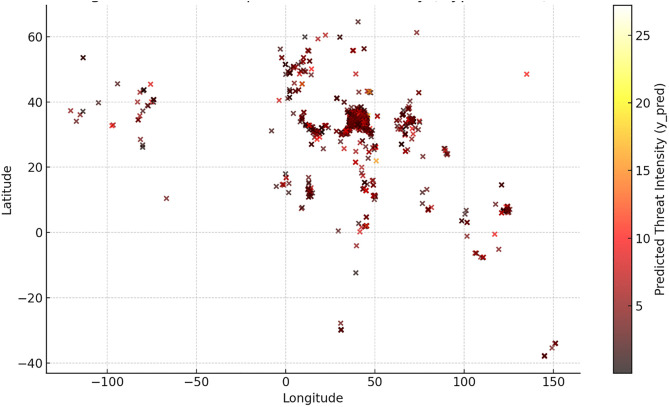
The geospatial distribution of estimated spatial intensity values. The figure shows a map of the expected degree of terrorism threat in the various world regions as obtained in the documented cases of ISIS affiliated attacks from 2012 up to 2019. Every individual mark show geolocated activity, the color gradient shows model approximations of the risk level. The visualization will assist in geospatial interpretation of model outputs and identify the possible urban vulnerable areas.

To validate spatial performance, the predicted risk intensity (y_pred) was visualized on a geographic map using interpolation and geohash-based binning. The geospatial distribution of predicted threat intensities is visualized in Fig 3 as follows:

Key observations: The resulting estimated predicted intensity patterns closely match the observed historical hotspots of ISIS, such as northern Iraq, eastern Syria and Libya, suggesting that the model accurately captures the spatial patterns of terrorist activity. We also note higher intensity patterns in neighbouring regions, indicating a capacity of the model to replicate larger spatial patterns beyond observed event points. Also, the findings reflect changes in the locations of the centres of intensity over time as ISIS expands and contracts its territories, suggesting responsiveness to spatial–temporal dynamics in the data.

### 4.6. Error analysis and robustness

The results of the residual tests indicate good uniformity in model performance over the study area. Other plots (residual plots not shown) indicate that most of the predicted values are within the + /- 10% range of the observed spatial risk intensities indicating that there is minimal overall deviation between model output and the actual values. Underprediction is also found to be minor in the most sparsely occupied regions, and it can be explained by the fact that there was not enough training data in these places. This is as expected of data-driven models that act under the circumstances of non-uniform spatial representation and does not significantly influence the capacity of the model to reflect larger patterns of terrorism risk.

Spatial autocorrelation was not explicitly modeled (e.g., via Moran’s I or spatial lag terms), yet the RF model still maintained high accuracy, suggesting strong implicit learning of spatial relationships.

Furthermore, the fixed-parameter model does not seem to be overfitted for regional clusters, as shown from cross-validation performance. This consistency was further verified by additional checks performed with shuffled and stratified partitions having the same fixed parameters.

### 4.7. Summary of findings

The findings indicate that the Random Forest model has consistent predictive accuracy and has low absolute error and consistent behavior on log-scaled error metrics. The spatial and temporal features are also meaningful in adding to the explanatory performance, and the temporal variables reflect the seasonal factors affecting the distribution of the terrorism risk. The resulting geospatial visualizations offer interpretable maps of estimated spatial intensity patterns, which coincides well with regions of ISIS action recorded in the past, which validates the appropriateness of Random Forest models to locational risk estimation, Although the overall performance is good, some small weaknesses are noted, such as the decreased performance in low-density areas, and the lack of explicit modelling of spatiotemporal autocorrelation. These restrictions emphasize the possibility of methodological improvement of the framework in further development.

## 5. Discussion

This paper gives evidence of the opportunities that GeoAI-powered Random Forest Regression presents when modeling and estimating the spatial distribution of the intensity of terrorism, with reference to ISIS-related incidents in the period between 2012–2019. The integration of spatial coordinates, temporal markers, and incident-specific attributes demonstrates how machine learning (ML) can augment traditional security analysis in geographic contexts.

### 5.1. Interpretation of results

Random Forest model performed well with a R^2^ of 0.84, which confirmed its capacity to address complicated non-linear relationships between the geospatial and temporal characteristics. The feature importance analysis showed that the most prominent predictors were longitude, latitude, and month- indicating that the geolocation and the periodicity of the attack are important aspects of knowing the patterns of attack intensity.

The values predicted for intensity can be viewed relative to patterns of concentration and not as a measure of risk. Values with larger magnitude reflect areas where aggregation of incidents was higher during the study period, while smaller values reflect less aggregation. These values cannot be interpreted as probabilities of future events but offer a relative spatial distribution of events in the past.

This aligns with spatial theories in terrorism studies, such as the cluster-based operational logic of insurgent groups [48] and such as seasonal strategic escalation, which may relate to religious or political calendars [49]. The spatial intensity map also aids in interpretation of the model as representing spatially concentrated activity in the past, typical of ISIS.

### 5.2. Alignment with existing literature

The findings resonate with prior applications of RF in environmental hazard modeling [50] and wildfire risk prediction, suggesting that the algorithm’s strength in modeling spatial heterogeneity translates well to security scenarios [51]. While most terrorism prediction models focus on event classification or actor profiling [52], our work emphasizes quantitative spatial intensity estimation, bridging the gap between ML prediction and GIS-based planning.

Further, by using geohash-based gridding and spatial visualization, this study adds to the growing body of work that emphasizes the operational interpretability of AI models for policymakers and urban planners [53].

### 5.3. Contributions to the field

This paper adds value to the existing body of literature in a number of ways. Methodologically, it illustrates the effectiveness of using geospatial data to incorporate the random forest regression in estimating spatial variations in terrorism risk and provides a viable solution to locational risk modelling. Explainability wise, feature importance analysis can give explainable insights about the behavior of the model which can then allow the analysts to correlate predictive outputs with real world spatial and contextual aspects. Spatially intensive maps produced by the model are useful products for exploratory purposes, and can be usefully applied in the context of a research scenario with limited resources for situational understanding in retrospect. The outputs might be of relevance to researchers in developing or resource-poor countries who can benefit from the relatively low cost of computation and spatial generalizability of the method for future exploratory or scenario analyses, pending additional validation prior to any operational application.

### 5.4. Practical implications

The modelling framework offers a systematic way of assessing spatial patterns of concentration of terrorism events. The outcomes may help exploratory spatial analysis through the identification of regions with relatively higher historic intensity, which could be helpful for comparative and contingency analysis.

The framework does not provide real-time analysis or forecasting, but it may assist in understanding past events and in identifying patterns. This might help researchers, planners and analysts understand the spatial patterns of historic events and to develop specific insights to the given context.

The low computational demands of the Random Forest model also indicate that such models can be applied in constrained settings for spatial analysis purposes, especially if the aim is to gain insight into historical patterns rather than predicting future events.

### 5.5. Limitations

Although the overall strength of the proposed approach is considerable, there are a few limitations that should be considered. First, the model fails to explicitly model the spatial autocorrelation structure, though the Random Forest may indirectly model the spatial effects by having correlated predictors, future research might need to explicitly model the spatial dependence with the use of a Moran I or a spatial lag formulation to reinforce the spatial inference. Although this study did not explicitly model spatial autocorrelation using statistical measures of spatial dependence such as Moran’s I, the spatial validation method used in this study offers an indirect measure of spatial dependence. Second, the evaluation is done on one main algorithm; as much as Random Forest is shown to be a reliable algorithm, a comparison with other ensemble algorithms like XGBoost or AdaBoost, and the current development of spatial deep learning [54] would be a way of gaining more information on relative performance and generalizability. Lastly, the existing implementation is based on static representations of features and does not use real-time or near-real-time sources of data, including social media cues or satellite-based data, which can be used to make the framework more temporal and adaptive in future applications. The model does not draw any conclusions about causal relationships at the incident level and it shouldn’t be used as a predictor of future terrorist attacks. It is rather a spatial intensity model based on past events.

### 5.6. Recommendations for future research

Several future research directions can be identified out of this research. It would be helpful to conduct comparative benchmarking of other ensemble techniques and neural network-based models on the same dataset to gain a better understanding of the relative strengths and weaknesses of various algorithmic methods in the same spatial and time context. Further research could investigate how to integrate dynamic data streams, and the adaptability of spatial intensity modelling frameworks for more responsive analytical applications. The generalizability of the suggested GeoAI framework also requires additional investigation through the application to other terrorist groups and conflict scenarios other than the ISIS-related ones. Lastly, further linkage with urban infrastructure data, including population density, transport systems, and land-use overlay, might make it possible to model secondary or collateral effects, which would make spatial risk estimation more relevant to urban resiliency and emergency management.

## 6. Conclusion

This paper has investigated the suitability of the Random Forest regression in a Geospatial Artificial Intelligence (GeoAI) context to the spatial distribution of ISIS/ISIL-related terrorist attacks between 2012 and 2019. The model that incorporated geospatial variables like longitude, latitude, and time features in a machine learning pipeline exhibited a good representation of spatial and temporal variation patterns in normalized incident intensity with a high level of explanatory performance (R^2^ = 0.84).

The findings can be used to visualize and spatially grid the differences in attack intensity in geographic units, as opposed to forecasting specific incidents or threats on real time, with the help of spatial gridding and visualization. The outputs offer a systematic, data-intensive foundation of investigating past concentration trends of terrorist operations, as well as, to aid in the exploratory spatial analysis of research and planning. The method illustrates how ensemble learning techniques can be used to supplement geospatial analysis in the context of working with complex and heterogeneous datasets on terrorism.

The results can be added to the emerging interplay between artificial intelligence and geographic information science by proving a scaling and explainable modeling framework of spatial intensity analysis. Nevertheless, the research is limited in several ways, such as the lack of clear treatment of spatial autocorrelation, work with other fixed historical data and the application of one modeling method. The limitations can be discussed in the future by benchmarking alternative models, adding dynamic data layers, and combining machine learning with spatial statistical methods.

In summary, this research shows the potential of GeoAI approaches for modelling spatial patterns of terrorism attacks based on historical data. The methodology offers a replicable and explainable method of modelling spatial intensity patterns, and adds to the understanding of the spatial distribution of terrorist incidents. Although the model is not suitable for real-time predictions, it provides a stepping-stone towards future research on spatial risk modelling and scenario analysis.
