# Supplementary material for: Spatial intensity modelling of ISIS-related attacks using random forest regression: A GeoAI-based analysis of GTD data (2012–2019)
Source: PLoS One. 2026 Jul 30;21(7):e0355100. doi: 10.1371/journal.pone.0355100 (PMC13422857; doi:10.1371/journal.pone.0355100)
Supplement: Attachment — Submitted filename: RESPONSE TO REVIEWERS 1 and 2 merged.pdf [file pone.0355100.s002.pdf]

RESPONSE TO REVIEWER #1

Reviewer #1 – General Comment

This manuscript presents a GeoAI-based framework… could make a valuable contribution…

Response:

We sincerely thank the reviewer for the positive and constructive evaluation of our manuscript. We greatly appreciate the recognition of the study's relevance to geospatial intelligence and spatial data science, as well as the acknowledgment of its structured methodology, reproducibility, and potential contribution to the field.

We have carefully considered all suggestions provided and have revised the manuscript accordingly to further improve methodological clarity, analytical rigor, and overall presentation. Our detailed responses are provided below.

1. Baseline Model Comparison

Reviewer Comment:

The manuscript would benefit from comparison with additional baseline models…

Response:

We agree with the reviewer that baseline comparison is essential for contextualizing model performance.

Accordingly, we have introduced a new subsection:

- **Section 4.1: Comparative Baseline Evaluation**

In this section, we include comparisons with:

- a naive mean baseline,
- a coordinate-only model (latitude and longitude), and
- a linear regression model.

The results demonstrate that the Random Forest model outperforms these baseline approaches, indicating that it captures additional structure beyond simple spatial location effects. This addition strengthens the comparative validity of the modelling framework.

2. Spatial Autocorrelation

Reviewer Comment:

Spatial autocorrelation is not explicitly modelled…

Response:

We thank the reviewer for this important observation.

To address this concern, we have incorporated an additional validation strategy:

- **Section 4.2: Spatial Validation and Robustness Assessment**

A spatial holdout validation approach was implemented, where observations are grouped by spatial units (grid cells/geohash regions), ensuring that all records from a given spatial unit are assigned exclusively to either the training or testing set. This prevents spatial leakage and provides a more realistic assessment of model performance under spatial independence conditions.

While explicit spatial statistical modelling techniques (e.g., Moran's I or spatial lag models) are not included in the current study, this has now been acknowledged as a limitation and identified as a direction for future research in Section 5.5 (Limitations).

3. Spatial Visualization Detail

Reviewer Comment:

More methodological detail is needed regarding interpolation and aggregation…

Response:

We appreciate this helpful suggestion.

We have expanded Section 3.5 (Spatial Visualization and Interpretation) to clarify the visualization process. Specifically, we now explain:

- the use of grid-based aggregation and geohash encoding for spatial grouping,
- that predicted values are visualized directly within discrete spatial units, and
- that no interpolation techniques were applied, in order to avoid introducing additional assumptions or artificial smoothing.

These additions improve methodological transparency and reproducibility.

4. Language and Style

Reviewer Comment: Grammatical *and stylistic issues…*

Response:

We fully agree with the reviewer and have conducted a comprehensive language revision of the manuscript.

This includes:

- correcting grammatical and syntactic issues,
- improving sentence clarity and flow, and
- standardizing terminology throughout the manuscript (e.g., consistent use of "modelling" and "spatial intensity estimation" instead of "prediction" or "forecasting" where appropriate).

These revisions significantly enhance readability and academic tone.

5. Interpretation of Intensity Values

Reviewer Comment: *Additional discussion on practical interpretation…*

Response:

We thank the reviewer for this valuable suggestion.

We have expanded the discussion in **Section 5.1 (Interpretation of Results)** to clarify the meaning of predicted intensity values. The revised text explicitly states that:

- the model outputs represent **relative spatial intensity patterns** derived from historical data,
- they do not correspond to probabilities of future events, and
- they should be interpreted as comparative indicators of spatial concentration rather than operational risk predictions.

This clarification improves accessibility for readers from non-technical backgrounds.

Final Comment

Response:

We sincerely thank the reviewer once again for the encouraging feedback and constructive suggestions. We believe that the revisions made in response to these comments have further strengthened the methodological rigor, clarity, and overall contribution of the manuscript. We hope that the revised version meets the expectations for publication.

RESPONSE TO REVIEWERS (Reviewer #2)

Reviewer #2 – General Comment

This study applies Random Forest regression/ requires major revision

Response:

We sincerely thank the reviewer for the detailed and technically insightful evaluation of our manuscript. The comments have been extremely valuable in improving the clarity, methodological transparency, and overall rigor of the study. We have carefully revised the manuscript to address all concerns raised, with substantial improvements made across the technical design, statistical validation, and presentation of results.

In particular, we have:

- clarified the modelling objective and unit of analysis,
- corrected inconsistencies related to hyperparameter tuning,
- introduced baseline model comparisons,
- implemented an additional spatial validation strategy to address potential performance inflation,
- and revised the manuscript extensively for clarity, terminology consistency, and academic tone.

All changes are described in detail below.

1. TECHNICAL SOUNDNESS

Reviewer Comment:

The most critical flaw is a unit-of-analysis mismatch… model learns location rather than relationship

Response:

We thank the reviewer for highlighting this important concern. We acknowledge that the initial presentation of the model may have led to ambiguity regarding the unit of analysis and modelling objective.

In the revised manuscript, we have explicitly clarified that the framework is designed to model relative spatial intensity patterns, rather than to establish incident-level causal relationships or predict individual events. The target variable represents a normalized aggregation of incident counts at the grid-cell level, and multiple incidents within the same spatial unit share the same intensity value by design.

To address this concern, we have:

- added a detailed clarification in Section 3.2.6 (Target Variable) explaining the relationship between incident-level features and spatially aggregated targets,
- reframed the study consistently throughout the manuscript as a spatial intensity modelling approach, rather than a predictive or forecasting model,
- removed or revised statements referring to prediction at "unknown locations" or real-time risk estimation.

These revisions ensure that the modelling framework is correctly interpreted as an exploratory spatial analysis tool rather than an event-level predictive model.

2. HYPERPARAMETER TUNING ISSUE

Reviewer Comment:

Hyperparameter tuning claim is not credible/ values are default

Response:

We appreciate the reviewer's careful observation. We acknowledge that the original manuscript incorrectly stated that hyperparameter tuning was performed via grid search.

This has been corrected in the revised manuscript. We now clarify that the Random Forest model was implemented using standard scikit-learn parameter settings. This approach was retained intentionally to ensure transparency, reproducibility, and consistency across comparative evaluations, in line with the exploratory objective of the study.

All references to hyperparameter tuning have been removed, and Section 3.3.1 has been revised accordingly.

3. STATISTICAL RIGOR (SPATIAL VALIDATION)

Reviewer Comment:

Random 80/20 split leads to performance inflation… spatial autocorrelation not addressed

Response:

We thank the reviewer for raising this critical point. To address the concern regarding potential performance inflation due to spatial autocorrelation, we have introduced an additional validation strategy in the revised manuscript.

Specifically, we implemented a spatial holdout validation approach, where observations were grouped by spatial units (grid cells/geohash regions), ensuring that all records from a given spatial unit were assigned exclusively to either the training or testing set. This prevents spatial leakage between training and test data.

The model was re-evaluated under this spatial validation framework, and the results are now reported in a new subsection:

- **Section 4.2: Spatial Validation and Robustness Assessment**

The updated results show:

- R^2 decreased from 0.84 (random split) to 0.69 (spatial holdout)
- RMSLE increased from 0.213 to 0.272
- MAE increased from 0.098 to 0.121

As expected, performance under spatial validation is lower, reflecting the influence of spatial dependence. However, the model retains meaningful explanatory capability, indicating that it captures broader spatial patterns beyond simple geographic memorization.

This addition significantly strengthens the statistical validity of the study.

4. BASELINE COMPARISON

Reviewer Comment:

No baseline model comparison provided…

Response:

We agree with the reviewer that baseline comparison is essential for evaluating model performance.

In the revised manuscript, we have added a new subsection:

- **Section 4.1: Comparative Baseline Evaluation**

We implemented multiple baseline models, including:

- a naive mean baseline,
- a coordinate-only model (using latitude and longitude only),
- and a linear regression model.

The results demonstrate that the Random Forest model outperforms these baselines, indicating that it captures additional structure beyond simple spatial location effects. At the same time, the coordinate-only model exhibits relatively strong performance, confirming the dominant influence of spatial dependence in the constructed target variable.

5. FEATURE IMPORTANCE INTERPRETATION

Reviewer Comment:

Latitude/longitude dominance is tautological

Response:

We thank the reviewer for this observation. We agree that the dominance of spatial coordinates is expected given the spatial construction of the target variable.

To address this, we have revised the interpretation in Section 4.2, explicitly noting that:

- feature importance results should be interpreted descriptively,
- and that the prominence of latitude and longitude reflects the spatial aggregation of the target variable rather than a causal relationship.

This clarification ensures that the results are not overstated.

6. PRESENTATION & LANGUAGE

Reviewer Comment:

Language issues, inconsistent terminology, unsupported claim

Response:

We appreciate the reviewer's feedback and have undertaken a comprehensive revision of the manuscript to improve clarity, consistency, and academic tone.

Specifically:

- all sections have been edited for grammatical accuracy and readability,
- terminology has been standardized (e.g., replacing "prediction" and "forecasting" with "modelling" or "estimation" where appropriate),
- unsupported claims related to early warning systems, real-time prediction, and deployment have been removed or carefully reframed,
- mismatched references have been reviewed and corrected to ensure relevance.

These revisions significantly improve the overall presentation and coherence of the manuscript.

CLOSING REMARKS

We sincerely thank the reviewer once again for their constructive and detailed feedback. We believe that the revisions made in response to these comments have substantially improved the methodological rigor, clarity, and contribution of the manuscript. We hope that the revised version meets the expectations for publication.
