# Supplementary material for: Spatial intensity modelling of ISIS-related attacks using random forest regression: A GeoAI-based analysis of GTD data (2012–2019)
Source: PLoS One. 2026 Jul 30;21(7):e0355100. doi: 10.1371/journal.pone.0355100 (PMC13422857; doi:10.1371/journal.pone.0355100)
Supplement: Attachment — Submitted filename: Response to Reviewers pdf 12 July.pdf [file pone.0355100.s004.pdf]

Response to Reviewers

Manuscript: PONE-D-26-06707R2

Title: Spatial Intensity Modelling of ISIS-Related Attacks Using Random Forest Regression: A GeoAI-Based Analysis of GTD Data (2012–2019)

Dear Dr. Li,

We thank the Guest Editor for the additional careful review of our manuscript and for the constructive comments provided. We have addressed each of the four points raised in the Additional Editor Comments below, along with the two Journal Requirements. All changes were made conservatively, preserving the original structure, methods, results, tables, and conclusions, and are limited to the specific sentences and table entries identified below. A point-by-point response follows.

1. Comparative Baselines

> *"Section 4.1 now includes a comparative table, but it does not address the specific baselines requested in the previous decision letter. Please add the naive mean baseline, coordinate-only model, and linear regression model, using the same metrics as the Random Forest model (R2, RMSLE, and MAE), or clearly justify any baseline that cannot be implemented."*

Response: We have added all three requested baselines to Table 2 (Section 4.1), evaluated using the same three metrics (R^2, RMSLE, MAE) as the other models in the comparison:

Naive Mean Baseline: $R^2 = 0.00$, RMSLE = 0.391, MAE = 0.188

Coordinate-Only Model: $R^2 = 0.41$, RMSLE = 0.338, MAE = 0.161

Linear Regression: $R^2 = 0.56$, RMSLE = 0.317, MAE = 0.149

These are presented alongside the existing Decision Tree, AdaBoost, XGBoost, and Random Forest results, in order of increasing model complexity. The introductory sentence to Section 4.1 and the closing interpretive paragraph were both updated to describe the full comparison set and the resulting performance progression.

2. Model Tuning

> *"Please remove the remaining inconsistent text stating that hyperparameters were "tuned via grid search" and reconcile all language with the stated use of fixed/default scikit-learn parameters and no additional hyperparameter optimization."*

Response: We confirm that the phrase "tuned via grid search" does not appear anywhere in the current manuscript text. To remove any remaining ambiguity, we revised two passages that referenced cross-validation in a way that could be misread as implying parameter selection:

In Section 4.2, the description of the training procedure now explicitly states that the fixed parameters in Table 1 were used throughout, and that 5-fold cross-validation was used only to assess the stability of model performance, not to select or tune parameters.

In Section 4.6, the description of the robustness checks was revised to explicitly tie the cross-validation and shuffled/stratified partition checks back to the same fixed-parameter configuration.

These edits ensure that every mention of cross-validation in the manuscript is consistent with the fixed-parameter, no-tuning approach described in Section 3.3.1.

3. Clean Manuscript and Numbering

> *"Please submit a clean version without tracked-change or formatting artifacts. The current PDF still appears to include residual artifacts such as "Formatted: Font: Not Bold", merged replacement text such as "estimatepredict", and inconsistent numbering such as "4.21" and "Table 32/Table 2"."*

Response: We reviewed the manuscript file directly at the XML level and confirmed that none of the specific artifacts described (residual "Formatted: Font" comments, the merged term "estimatepredict", section numbering such as "4.21", or duplicated table numbering such as "Table 32/Table 2") are present in the current file. Section numbering runs sequentially from 4.1 through 4.7, and all five tables are numbered sequentially with no duplication.

During this review we did identify and correct a small number of leftover punctuation artifacts (isolated duplicate periods) introduced by the editing process itself. These have been removed. All tracked changes have been accepted in the clean "Manuscript" file submitted alongside this letter, and a separate "Revised Manuscript with Track Changes" file is provided showing the full history of edits for this and the previous revision round.

4. Scope of Claims

> *"Please continue to frame the work as retrospective spatial intensity modeling based on historical data. Any remaining language that suggests forecasting, real-time prediction, operational deployment, or prescriptive risk assessment should be removed or clearly limited as future work beyond the current analysis."*

Response: We revised eight passages across Sections 1, 2, 3, 3.1, 3.5, 4.5, and 5.3 that used language implying operational deployment, prescriptive planning, or active forecasting capability. In each case the wording was replaced with language framing the work as retrospective, exploratory spatial intensity analysis. Specific changes include:

Section 1: Removed language suggesting the model output could be used by "planners and law enforcement organizations" to "prioritize resource allocation" and perform "risk-based assessments," replacing it with a description of the visualizations as a retrospective reference point for future exploratory analysis.

Section 1: Removed the phrase "actionable intelligence to the stakeholders of an urban security plan," replacing it with language describing the contribution as supporting retrospective, exploratory understanding for researchers and analysts.

Section 2: Revised language describing the framework as informing "security operations" and bridging "decision support and predictive analytics in counter-terrorism planning," replacing it with a description of the work as a repeatable GeoAI process for retrospective spatial intensity modelling.

Section 3 (Methodology introduction) and Section 3.1: Replaced references to a "prediction model" with "spatial intensity model" and removed the phrase "operationally significant scope," replacing it with "clearly defined and analytically tractable scope."

Section 3.5: Revised language describing the visualization workflow as filling "the gap between predictive modelling and practical applicability" and enabling "strategic planning," replacing it with language describing the visualizations as supporting exploratory interpretation of the modelling results. Similarly revised a passage describing the findings as making the model "more actionable" for "real-life plans associated with counterterrorism and urban security planning,"

replacing it with language describing the visualization as supporting exploratory interpretation rather than operational planning use.

Section 4.5: Revised the Figure 3 caption from “The geospatial distribution of predicted threat intensities” to “The geospatial distribution of estimated spatial intensity values,” for consistency with the retrospective framing used elsewhere.

Section 5.3: Revised the passage describing the model as supporting “situational awareness and resource distribution in the high-risk urban setting” and “augmenting proactive security planning and reducing operational cost,” replacing it with language describing the outputs as useful for exploratory, retrospective purposes in resource-constrained research contexts, explicitly noting that any operational use would require further validation.

Sections 5.4 through 5.6 and the Conclusion already framed the work appropriately as retrospective and exploratory in the previous revision and were left unchanged.

Journal Requirements

1. Citations recommended by reviewers: No specific additional citations were recommended by the editor in this decision letter beyond the four numbered comments addressed above; accordingly, no changes were made to the reference list on this basis.

2. Reference list completeness: We reviewed the reference list and confirm that it is complete and correct, and that it does not include any retracted publications.

We thank the Guest Editor again for the guidance provided through this review process and believe the revised manuscript now fully addresses the outstanding concerns. We look forward to the Editor's further consideration.

Sincerely,

Dr. R.G.N. Lakmali
On behalf of all authors
