# Supplementary material for: Spatial intensity modelling of ISIS-related attacks using random forest regression: A GeoAI-based analysis of GTD data (2012–2019)
Source: PLoS One. 2026 Jul 30;21(7):e0355100. doi: 10.1371/journal.pone.0355100 (PMC13422857; doi:10.1371/journal.pone.0355100)
Supplement: Attachment — Submitted filename: Response to Aditional Reviewer 08 June 2026.docx [file pone.0355100.s003.docx]

Manuscript ID: PONE-D-26-06707R1

Title: Spatial Intensity Modelling of ISIS-Related Attacks Using Random Forest Regression: A GeoAI-Based Analysis of GTD Data (2012–2019)

**Response to Additional Editorial Comments**

We sincerely thank the Editor for the careful review of our revised manuscript and for the constructive observations provided. We have carefully considered all comments and revised the manuscript accordingly. Our responses are provided below.

Comment 1:

The response letter states that a new Section 4.1, "Comparative Baseline Evaluation," was added with comparisons against a naive mean baseline, a coordinate-only model, and a linear regression model. I could not locate this section or these baseline results in the revised manuscript. Please add the baseline comparison results clearly in the manuscript, including the same evaluation metrics used for the Random Forest model.

Response:

Thank you for this observation. A dedicated subsection entitled "4.1 Comparative Baseline Evaluation" has now been included in the manuscript. Comparative model evaluation results have been added and reported using the same evaluation metrics (R², RMSLE and MAE) used for the Random Forest model. The purpose of this section is to provide additional context regarding model performance and support the selection of Random Forest as the primary modelling framework.

Comment 2:

Please ensure consistency regarding model tuning. Some text still appears to refer to grid search or hyperparameter tuning, while other parts state that default scikit-learn parameters were used and no tuning was conducted. These statements should be reconciled throughout the manuscript.

Response:

We appreciate this observation. The manuscript has been thoroughly reviewed to ensure consistency regarding model implementation. All references to grid search, hyperparameter tuning and optimisation procedures have been reconciled with the actual modelling process. The revised manuscript consistently states that fixed model parameters were used throughout the analysis and that no additional hyperparameter optimisation was performed.

Comment 3:

Please submit a clean revised manuscript without formatting or tracked-change artifacts. The current file appears to contain residual formatting markers and duplicated or inconsistent section/table numbering.

Response:

This issue has been addressed. A clean revised manuscript has been prepared and submitted. Formatting artefacts, duplicated numbering, and section/table numbering inconsistencies have been corrected. All section headings, table references, figure references and numbering sequences have been reviewed and standardised throughout the manuscript.

Comment 4:

Please continue to frame the study as retrospective spatial intensity modelling rather than real-time forecasting or operational prediction and remove any remaining claims that imply early-warning or deployment-ready predictive use unless they are directly supported by the analysis.

Response:

Thank you for this important observation. The manuscript has undergone a further review to ensure consistent framing of the study as a retrospective spatial intensity modelling exercise based on historical ISIS/ISIL incident data. Additional revisions were made throughout the manuscript to remove or modify terminology that could imply real-time forecasting, operational prediction, early-warning functionality or deployment-ready applications. The revised manuscript now consistently presents the framework as a historical spatial intensity modelling approach intended for exploratory spatial analysis and scenario-based learning.

We sincerely thank the Editor for the valuable guidance provided during the revision process. The additional comments have significantly improved the clarity, consistency and presentation of the manuscript, and we hope that the revised version will now be suitable for further consideration.

Dr. Rubasin Gamage Niluka Lakmali
Corresponding Author
On behalf of all authors
