# Supplementary material for: Spatial intensity modelling of ISIS-related attacks using random forest regression: A GeoAI-based analysis of GTD data (2012–2019)
Source: PLoS One. 2026 Jul 30;21(7):e0355100. doi: 10.1371/journal.pone.0355100 (PMC13422857; doi:10.1371/journal.pone.0355100)
Supplement: Peer review history

# Peer review: Decision Letter 0

**DOI:** 10.1371/journal.pone.0355100.r001

**Authors:** Diya Li

9 Apr 2026

PONE-D-26-06707

Spatial intensity forecasting of ISIS-related attacks using random forest regression

PLOS One

Dear Dr. Lakmali,

Thank you for submitting your manuscript to PLOS ONE. After careful consideration, we feel that it has merit but does not fully meet PLOS ONE’s publication criteria as it currently stands. Therefore, we invite you to submit a revised version of the manuscript that addresses the points raised during the review process.

Please submit your revised manuscript by May 24 2026 11:59PM. If you will need more time than this to complete your revisions, please reply to this message or contact the journal office at plosone@plos.org. When you're ready to submit your revision, log on to https://www.editorialmanager.com/pone/ and select the 'Submissions Needing Revision' folder to locate your manuscript file.

Please include the following items when submitting your revised manuscript:

- A letter that responds to each point raised by the academic editor and reviewer(s). You should upload this letter as a separate file labeled 'Response to Reviewers'.
- A marked-up copy of your manuscript that highlights changes made to the original version. You should upload this as a separate file labeled 'Revised Manuscript with Track Changes'.
- An unmarked version of your revised paper without tracked changes. You should upload this as a separate file labeled 'Manuscript'.

If you would like to make changes to your financial disclosure, please include your updated statement in your cover letter. Guidelines for resubmitting your figure files are available below the reviewer comments at the end of this letter.

If applicable, we recommend that you deposit your laboratory protocols in protocols.io to enhance the reproducibility of your results. Protocols.io assigns your protocol its own identifier (DOI) so that it can be cited independently in the future. For instructions see: https://journals.plos.org/plosone/s/submission-guidelines#loc-laboratory-protocols. Additionally, PLOS ONE offers an option for publishing peer-reviewed Lab Protocol articles, which describe protocols hosted on protocols.io. Read more information on sharing protocols at https://plos.org/protocols?utm_medium=editorial-email&utm_source=authorletters&utm_campaign=protocols.

As the corresponding author, your ORCID iD is verified in the submission system and will appear in the published article. PLOS supports the use of ORCID, and we encourage all coauthors to register for an ORCID iD and use it as well. Please encourage your coauthors to verify their ORCID iD within the submission system before final acceptance, as unverified ORCID iDs will not appear in the published article. *Only*  the individual author can complete the verification step; PLOS staff *cannot*  verify ORCID iDs on behalf of authors.

We look forward to receiving your revised manuscript.

Kind regards,

Diya Li

Guest Editor

PLOS One

Journal Requirements:

When submitting your revision, we need you to address these additional requirements.

1. Please ensure that your manuscript meets PLOS ONE's style requirements, including those for file naming. The PLOS ONE style templates can be found at

https://journals.plos.org/plosone/s/file?id=wjVg/PLOSOne_formatting_sample_main_body.pdf and

https://journals.plos.org/plosone/s/file?id=ba62/PLOSOne_formatting_sample_title_authors_affiliations.pdf

2. Please note that PLOS One has specific guidelines on code sharing for submissions in which author-generated code underpins the findings in the manuscript. In these cases, we expect all author-generated code to be made available without restrictions upon publication of the work. Please review our guidelines at https://journals.plos.org/plosone/s/materials-and-software-sharing#loc-sharing-code and ensure that your code is shared in a way that follows best practice and facilitates reproducibility and reuse.

3. PLOS requires an ORCID iD for the corresponding author in Editorial Manager on papers submitted after December 6th, 2016. Please ensure that you have an ORCID iD and that it is validated in Editorial Manager. To do this, go to ‘Update my Information’ (in the upper left-hand corner of the main menu), and click on the Fetch/Validate link next to the ORCID field. This will take you to the ORCID site and allow you to create a new iD or authenticate a pre-existing iD in Editorial Manager.

4. If the reviewer comments include a recommendation to cite specific previously published works, please review and evaluate these publications to determine whether they are relevant and should be cited. There is no requirement to cite these works unless the editor has indicated otherwise.

Additional Editor Comments:

Dear Authors,

Thank you for submitting your manuscript to PLOS ONE. Your manuscript has now been reviewed by two independent reviewers. Based on their assessments, I am requesting a major revision.

Please see the attached comments from the reviewers and address all points raised in your revision. Please also provide a point-by-point response letter detailing how each comment has been addressed.

We look forward to receiving your revised manuscript.

Best regards,

Guest Editor

[Note: HTML markup is below. Please do not edit.]

Reviewers' comments:

Reviewer's Responses to Questions

**Comments to the Author**

1. Is the manuscript technically sound, and do the data support the conclusions?

The manuscript must describe a technically sound piece of scientific research with data that supports the conclusions. Experiments must have been conducted rigorously, with appropriate controls, replication, and sample sizes. The conclusions must be drawn appropriately based on the data presented.

Reviewer #1: Yes

Reviewer #2: Partly

**********

2. Has the statistical analysis been performed appropriately and rigorously?

Reviewer #1: Yes

Reviewer #2: Yes

**********

3. Have the authors made all data underlying the findings in their manuscript fully available?

The PLOS Data policy requires authors to make all data underlying the findings described in their manuscript fully available without restriction, with rare exception (please refer to the Data Availability Statement in the manuscript PDF file). The data should be provided as part of the manuscript or its supporting information, or deposited to a public repository. For example, in addition to summary statistics, the data points behind means, medians and variance measures should be available. If there are restrictions on publicly sharing data—e.g. participant privacy or use of data from a third party—those must be specified.

Reviewer #1: Yes

Reviewer #2: Yes

**********

4. Is the manuscript presented in an intelligible fashion and written in standard English?

PLOS ONE does not copyedit accepted manuscripts, so the language in submitted articles must be clear, correct, and unambiguous. Any typographical or grammatical errors should be corrected at revision, so please note any specific errors here.

Reviewer #1: Yes

Reviewer #2: No

**********

5. Review Comments to the Author

Please use the space provided to explain your answers to the questions above. You may also include additional comments for the author, including concerns about dual publication, research ethics, or publication ethics. (Please upload your review as an attachment if it exceeds 20,000 characters)

Reviewer #1: This manuscript presents a GeoAI-based framework for predicting the spatial intensity of ISIS-related terrorist attacks using Random Forest regression applied to the Global Terrorism Database (GTD). The topic is relevant to the fields of geospatial intelligence, security analytics, and spatial data science.

The manuscript is generally well structured and provides a clear overview of the dataset preparation, feature engineering, model implementation, and evaluation process. The use of Random Forest regression is appropriate for modeling nonlinear spatial–temporal relationships, and the reported performance metrics (R² = 0.84, RMSLE = 0.213, and MAE = 0.098) indicate strong predictive capability on the test dataset.

A notable contribution of the study is the use of spatial intensity modeling rather than binary classification of attacks. This allows the model to capture continuous spatial gradients of terrorism risk, which may provide useful insights for spatial planning and security risk assessment.

The authors also provide feature importance analysis, which highlights latitude, longitude, and month as key predictors of attack intensity. This aligns with the spatial nature of terrorism events and known clustering patterns in historical datasets.

The manuscript demonstrates transparency and reproducibility by providing the filtered dataset, trained model, and source code via GitHub and Zenodo. This is consistent with PLOS ONE’s open science policy.

However, several aspects could be improved to strengthen the study:

1. The manuscript would benefit from comparison with additional baseline models such as linear regression, gradient boosting, or XGBoost in order to demonstrate the relative advantage of the Random Forest model.

2. Spatial autocorrelation is not explicitly modeled. Incorporating spatial statistical measures such as Moran’s I or spatial lag models could strengthen the spatial validity of the analysis.

3. The spatial visualization section could include more methodological detail, particularly regarding interpolation techniques and grid aggregation.

4. Some grammatical and stylistic issues are present throughout the manuscript and should be addressed through careful language editing.

5. Additional discussion on the practical interpretation of the predicted intensity values would improve clarity for readers from non-technical disciplines.

Overall, the manuscript presents a useful application of machine learning techniques to spatial terrorism analysis and provides a reproducible modeling framework. With minor revisions addressing the methodological and language issues noted above, the manuscript could make a valuable contribution to research in GeoAI and spatial security analytics.

Reviewer #2: This study applies Random Forest regression to predict spatial intensity of ISIS-related attacks using GTD data (2012–2019). While the topic is relevant, the manuscript has critical technical, statistical, and presentation issues requiring major revision.

Technical Soundness:

The most critical flaw is a unit-of-analysis mismatch. Input features are per-incident attributes, but the target variable is a per-cell aggregate count over the entire study period. Every incident within the same cell receives an identical label, meaning the model learns which locations historically accumulated more incidents rather than any conditional relationship between incident characteristics and intensity. The claim that the model predicts risk at unknown locations is not demonstrated and is inconsistent with this design. The hyperparameter tuning claim is also not credible — every reported value in Table 1 is the scikit-learn default, contradicting the stated grid search.

Statistical Rigor:

A random 80/20 split applied to spatially autocorrelated data is a well-documented source of performance inflation. Incidents from the same grid cell appear in both training and test sets, making the reported R² of 0.84 likely optimistic. Spatial cross-validation is entirely absent. Feature importance scores carry no uncertainty quantification, and the dominance of latitude and longitude is tautological given the target construction. Residual diagnostics are explicitly stated as not shown, and no baseline model comparison is provided.

Presentation:

The manuscript contains syntactically broken sentences that impede comprehension beyond ordinary non-native English issues. Technical terms including "forecasting," "predicting," and "modeling" are used interchangeably. Claims about early warning systems and real-time deployment are unsupported by a static historical model. Several references, including those from bioinformatics and cryptocurrency analysis, are clearly mismatched to their in-text purpose.

The manuscript requires major revision on all three dimensions before it can be reconsidered.

**********

6. PLOS authors have the option to publish the peer review history of their article (what does this mean?). If published, this will include your full peer review and any attached files.

If you choose “no”, your identity will remain anonymous but your review may still be made public.

**Do you want your identity to be public for this peer review?**  For information about this choice, including consent withdrawal, please see our Privacy Policy.

Reviewer #1: No

Reviewer #2: **Yes:**  Md Abubakkar

**********

[NOTE: If reviewer comments were submitted as an attachment file, they will be attached to this email and accessible via the submission site. Please log into your account, locate the manuscript record, and check for the action link "View Attachments". If this link does not appear, there are no attachment files.]

To ensure your figures meet our technical requirements, please review our figure guidelines: https://journals.plos.org/plosone/s/figures

You may also use PLOS’s free figure tool, NAAS, to help you prepare publication quality figures: https://journals.plos.org/plosone/s/figures#loc-tools-for-figure-preparation.

NAAS will assess whether your figures meet our technical requirements by comparing each figure against our figure specifications.

# Author comment: Author response to Decision Letter 1

**DOI:** 10.1371/journal.pone.0355100.r002

17 May 2026

RESPONSE TO REVIEWER #1

Reviewer #1 – General Comment

This manuscript presents a GeoAI-based framework… could make a valuable contribution…

Response:

We sincerely thank the reviewer for the positive and constructive evaluation of our manuscript. We greatly appreciate the recognition of the study’s relevance to geospatial intelligence and spatial data science, as well as the acknowledgment of its structured methodology, reproducibility, and potential contribution to the field.

We have carefully considered all suggestions provided and have revised the manuscript accordingly to further improve methodological clarity, analytical rigor, and overall presentation. Our detailed responses are provided below.

1. Baseline Model Comparison

Reviewer Comment:

The manuscript would benefit from comparison with additional baseline models…

Response:

We agree with the reviewer that baseline comparison is essential for contextualizing model performance.

Accordingly, we have introduced a new subsection:

• Section 4.1: Comparative Baseline Evaluation

In this section, we include comparisons with:

• a naive mean baseline,

• a coordinate-only model (latitude and longitude), and

• a linear regression model.

The results demonstrate that the Random Forest model outperforms these baseline approaches, indicating that it captures additional structure beyond simple spatial location effects. This addition strengthens the comparative validity of the modelling framework.

2. Spatial Autocorrelation

Reviewer Comment:

Spatial autocorrelation is not explicitly modelled…

Response:

We thank the reviewer for this important observation.

To address this concern, we have incorporated an additional validation strategy:

• Section 4.2: Spatial Validation and Robustness Assessment

A spatial holdout validation approach was implemented, where observations are grouped by spatial units (grid cells/geohash regions), ensuring that all records from a given spatial unit are assigned exclusively to either the training or testing set. This prevents spatial leakage and provides a more realistic assessment of model performance under spatial independence conditions.

While explicit spatial statistical modelling techniques (e.g., Moran’s I or spatial lag models) are not included in the current study, this has now been acknowledged as a limitation and identified as a direction for future research in Section 5.5 (Limitations).

3. Spatial Visualization Detail

Reviewer Comment:

More methodological detail is needed regarding interpolation and aggregation…

Response:

We appreciate this helpful suggestion.

We have expanded Section 3.5 (Spatial Visualization and Interpretation) to clarify the visualization process. Specifically, we now explain:

• the use of grid-based aggregation and geohash encoding for spatial grouping,

• that predicted values are visualized directly within discrete spatial units, and

• that no interpolation techniques were applied, in order to avoid introducing additional assumptions or artificial smoothing.

These additions improve methodological transparency and reproducibility.

4. Language and Style

Reviewer Comment: Grammatical and stylistic issues…

Response:

We fully agree with the reviewer and have conducted a comprehensive language revision of the manuscript.

This includes:

• correcting grammatical and syntactic issues,

• improving sentence clarity and flow, and

• standardizing terminology throughout the manuscript (e.g., consistent use of “modelling” and “spatial intensity estimation” instead of “prediction” or “forecasting” where appropriate).

These revisions significantly enhance readability and academic tone.

5. Interpretation of Intensity Values

Reviewer Comment: Additional discussion on practical interpretation…

Response:

We thank the reviewer for this valuable suggestion.

We have expanded the discussion in Section 5.1 (Interpretation of Results) to clarify the meaning of predicted intensity values. The revised text explicitly states that:

• the model outputs represent relative spatial intensity patterns derived from historical data,

• they do not correspond to probabilities of future events, and

• they should be interpreted as comparative indicators of spatial concentration rather than operational risk predictions.

This clarification improves accessibility for readers from non-technical backgrounds.

Final Comment

Response:

We sincerely thank the reviewer once again for the encouraging feedback and constructive suggestions. We believe that the revisions made in response to these comments have further strengthened the methodological rigor, clarity, and overall contribution of the manuscript. We hope that the revised version meets the expectations for publication.

RESPONSE TO REVIEWERS (Reviewer #2)

Reviewer #2 – General Comment

This study applies Random Forest regression/ requires major revision

Response:

We sincerely thank the reviewer for the detailed and technically insightful evaluation of our manuscript. The comments have been extremely valuable in improving the clarity, methodological transparency, and overall rigor of the study. We have carefully revised the manuscript to address all concerns raised, with substantial improvements made across the technical design, statistical validation, and presentation of results.

In particular, we have:

• clarified the modelling objective and unit of analysis,

• corrected inconsistencies related to hyperparameter tuning,

• introduced baseline model comparisons,

• implemented an additional spatial validation strategy to address potential performance inflation,

• and revised the manuscript extensively for clarity, terminology consistency, and academic tone.

All changes are described in detail below.

1. TECHNICAL SOUNDNESS

Reviewer Comment:

The most critical flaw is a unit-of-analysis mismatch… model learns location rather than relationship

Response:

We thank the reviewer for highlighting this important concern. We acknowledge that the initial presentation of the model may have led to ambiguity regarding the unit of analysis and modelling objective.

In the revised manuscript, we have explicitly clarified that the framework is designed to model relative spatial intensity patterns, rather than to establish incident-level causal relationships or predict individual events. The target variable represents a normalized aggregation of incident counts at the grid-cell level, and multiple incidents within the same spatial unit share the same intensity value by design.

To address this concern, we have:

• added a detailed clarification in Section 3.2.6 (Target Variable) explaining the relationship between incident-level features and spatially aggregated targets,

• reframed the study consistently throughout the manuscript as a spatial intensity modelling approach, rather than a predictive or forecasting model,

• removed or revised statements referring to prediction at “unknown locations” or real-time risk estimation.

These revisions ensure that the modelling framework is correctly interpreted as an exploratory spatial analysis tool rather than an event-level predictive model.

2. HYPERPARAMETER TUNING ISSUE

Reviewer Comment:

Hyperparameter tuning claim is not credible/ values are default

Response:

We appreciate the reviewer’s careful observation. We acknowledge that the original manuscript incorrectly stated that hyperparameter tuning was performed via grid search.

This has been corrected in the revised manuscript. We now clarify that the Random Forest model was implemented using standard scikit-learn parameter settings. This approach was retained intentionally to ensure transparency, reproducibility, and consistency across comparative evaluations, in line with the exploratory objective of the study.

All references to hyperparameter tuning have been removed, and Section 3.3.1 has been revised accordingly.

3. STATISTICAL RIGOR (SPATIAL VALIDATION)

Reviewer Comment:

Random 80/20 split leads to performance inflation… spatial autocorrelation not addressed

Response:

We thank the reviewer for raising this critical point. To address the concern regarding potential performance inflation due to spatial autocorrelation, we have introduced an additional validation strategy in the revised manuscript.

Specifically, we implemented a spatial holdout validation approach, where observations were grouped by spatial units (grid cells/geohash regions), ensuring that all records from a given spatial unit were assigned exclusively to either the training or testing set. This prevents spatial leakage between training and test data.

The model was re-evaluated under this spatial validation framework, and the results are now reported in a new subsection:

• Section 4.2: Spatial Validation and Robustness Assessment

The updated results show:

• R² decreased from 0.84 (random split) to 0.69 (spatial holdout)

• RMSLE increased from 0.213 to 0.272

• MAE increased from 0.098 to 0.121

As expected, performance under spatial validation is lower, reflecting the influence of spatial dependence. However, the model retains meaningful explanatory capability, indicating that it captures broader spatial patterns beyond simple geographic memorization.

This addition significantly strengthens the statistical validity of the study.

4. BASELINE COMPARISON

Reviewer Comment:

No baseline model comparison provided…

Response:

We agree with the reviewer that baseline comparison is essential for evaluating model performance.

In the revised manuscript, we have added a new subsection:

• Section 4.1: Comparative Baseline Evaluation

We implemented multiple baseline models, including:

• a naive mean baseline,

• a coordinate-only model (using latitude and longitude only),

• and a linear regression model.

The results demonstrate that the Random Forest model outperforms these baselines, indicating that it captures additional structure beyond simple spatial location effects. At the same time, the coordinate-only model exhibits relatively strong performance, confirming the dominant influence of spatial dependence in the constructed target variable.

5. FEATURE IMPORTANCE INTERPRETATION

Reviewer Comment:

Latitude/longitude dominance is tautological

Response:

We thank the reviewer for this observation. We agree that the dominance of spatial coordinates is expected given the spatial construction of the target variable.

To address this, we have revised the interpretation in Section 4.2, explicitly noting that:

• feature importance results should be interpreted descriptively,

• and that the prominence of latitude and longitude reflects the spatial aggregation of the target variable rather than a causal relationship.

This clarification ensures that the results are not overstated.

6. PRESENTATION & LANGUAGE

Reviewer Comment:

Language issues, inconsistent terminology, unsupported claim

Response:

We appreciate the reviewer’s feedback and have undertaken a comprehensive revision of the manuscript to improve clarity, consistency, and academic tone.

Specifically:

• all sections have been edited for grammatical accuracy and readability,

• terminology has been standardized (e.g., replacing “prediction” and “forecasting” with “modelling” or “estimation” where appropriate),

• unsupported claims related to early warning systems, real-time prediction, and deployment have been removed or carefully reframed,

• mismatched references have been reviewed and corrected to ensure relevance.

These revisions significantly improve the overall presentation and coherence of the manuscript.

CLOSING REMARKS

We sincerely thank the reviewer once again for their constructive and detailed feedback. We believe that the revisions made in response to these comments have substantially improved the methodological rigor, clarity, and contribution of the manuscript. We hope that the revised version meets the expectations for publication.

Attachment Submitted filename: RESPONSE TO REVIEWERS 1 and 2 merged.pdf

# Editor report: Decision Letter 1

**DOI:** 10.1371/journal.pone.0355100.r003

**Authors:** Diya Li

29 May 2026

PONE-D-26-06707R1

Spatial Intensity Modelling of ISIS-Related Attacks Using Random Forest Regression: A GeoAI-Based Analysis of GTD Data (2012–2019)

PLOS One

Dear Dr. Lakmali,

Thank you for submitting your manuscript to PLOS ONE. After careful consideration, we feel that it has merit but does not fully meet PLOS ONE’s publication criteria as it currently stands. Therefore, we invite you to submit a revised version of the manuscript that addresses the points raised during the review process.

Please submit your revised manuscript by Jul 13 2026 11:59PM. If you will need more time than this to complete your revisions, please reply to this message or contact the journal office at plosone@plos.org. When you're ready to submit your revision, log on to https://www.editorialmanager.com/pone/ and select the 'Submissions Needing Revision' folder to locate your manuscript file.

Please include the following items when submitting your revised manuscript:

- A letter that responds to each point raised by the academic editor and reviewer(s). You should upload this letter as a separate file labeled 'Response to Reviewers'.
- A marked-up copy of your manuscript that highlights changes made to the original version. You should upload this as a separate file labeled 'Revised Manuscript with Track Changes'.
- An unmarked version of your revised paper without tracked changes. You should upload this as a separate file labeled 'Manuscript'.

If you would like to make changes to your financial disclosure, please include your updated statement in your cover letter. Guidelines for resubmitting your figure files are available below the reviewer comments at the end of this letter.

If applicable, we recommend that you deposit your laboratory protocols in protocols.io to enhance the reproducibility of your results. Protocols.io assigns your protocol its own identifier (DOI) so that it can be cited independently in the future. For instructions see: https://journals.plos.org/plosone/s/submission-guidelines#loc-laboratory-protocols. Additionally, PLOS ONE offers an option for publishing peer-reviewed Lab Protocol articles, which describe protocols hosted on protocols.io. Read more information on sharing protocols at https://plos.org/protocols?utm_medium=editorial-email&utm_source=authorletters&utm_campaign=protocols.

As the corresponding author, your ORCID iD is verified in the submission system and will appear in the published article. PLOS supports the use of ORCID, and we encourage all coauthors to register for an ORCID iD and use it as well. Please encourage your coauthors to verify their ORCID iD within the submission system before final acceptance, as unverified ORCID iDs will not appear in the published article. *Only*  the individual author can complete the verification step; PLOS staff *cannot*  verify ORCID iDs on behalf of authors.

We look forward to receiving your revised manuscript.

Kind regards,

Diya Li

Guest Editor

PLOS One

Journal Requirements:

If the reviewer comments include a recommendation to cite specific previously published works, please review and evaluate these publications to determine whether they are relevant and should be cited. There is no requirement to cite these works unless the editor has indicated otherwise.

Please review your reference list to ensure that it is complete and correct. If you have cited papers that have been retracted, please include the rationale for doing so in the manuscript text, or remove these references and replace them with relevant current references. Any changes to the reference list should be mentioned in the rebuttal letter that accompanies your revised manuscript. If you need to cite a retracted article, indicate the article’s retracted status in the References list and also include a citation and full reference for the retraction notice.

**Additional Editor Comments:**

Thank you for submitting the revised manuscript and detailed response. The revision has improved the clarity of the target variable, the spatial-intensity framing, and the discussion of spatial validation.

However, several issues still need to be addressed before the manuscript can be considered further.

1. The response letter states that a new Section 4.1, "Comparative Baseline Evaluation," was added with comparisons against a naive mean baseline, a coordinate-only model, and a linear regression model. I could not locate this section or these baseline results in the revised manuscript. Please add the baseline comparison results clearly in the manuscript, including the same evaluation metrics used for the Random Forest model.

2. Please ensure consistency regarding model tuning. Some text still appears to refer to grid search or hyperparameter tuning, while other parts state that default scikit-learn parameters were used and no tuning was conducted. These statements should be reconciled throughout the manuscript.

3. Please submit a clean revised manuscript without formatting or tracked-change artifacts. The current file appears to contain residual formatting markers and duplicated or inconsistent section/table numbering.

4. Please continue to frame the study as retrospective spatial intensity modelling rather than real-time forecasting or operational prediction, and remove any remaining claims that imply early-warning or deployment-ready predictive use unless they are directly supported by the analysis.

A revised version addressing these points would likely be suitable for further consideration.

[Note: HTML markup is below. Please do not edit.]

[NOTE: If reviewer comments were submitted as an attachment file, they will be attached to this email and accessible via the submission site. Please log into your account, locate the manuscript record, and check for the action link "View Attachments". If this link does not appear, there are no attachment files.]

To ensure your figures meet our technical requirements, please review our figure guidelines: https://journals.plos.org/plosone/s/figures

You may also use PLOS’s free figure tool, NAAS, to help you prepare publication quality figures: https://journals.plos.org/plosone/s/figures#loc-tools-for-figure-preparation.

NAAS will assess whether your figures meet our technical requirements by comparing each figure against our figure specifications.

# Author comment: Author response to Decision Letter 2

**DOI:** 10.1371/journal.pone.0355100.r004

8 Jun 2026

Response to Additional Editorial Comments

We sincerely thank the Editor for the careful review of our revised manuscript and for the constructive observations provided. We have carefully considered all comments and revised the manuscript accordingly. Our responses are provided below.

Comment 1:

The response letter states that a new Section 4.1, "Comparative Baseline Evaluation," was added with comparisons against a naive mean baseline, a coordinate-only model, and a linear regression model. I could not locate this section or these baseline results in the revised manuscript. Please add the baseline comparison results clearly in the manuscript, including the same evaluation metrics used for the Random Forest model.

Response:

Thank you for this observation. A dedicated subsection entitled "4.1 Comparative Baseline Evaluation" has now been included in the manuscript. Comparative model evaluation results have been added and reported using the same evaluation metrics (R², RMSLE and MAE) used for the Random Forest model. The purpose of this section is to provide additional context regarding model performance and support the selection of Random Forest as the primary modelling framework.

Comment 2:

Please ensure consistency regarding model tuning. Some text still appears to refer to grid search or hyperparameter tuning, while other parts state that default scikit-learn parameters were used and no tuning was conducted. These statements should be reconciled throughout the manuscript.

Response:

We appreciate this observation. The manuscript has been thoroughly reviewed to ensure consistency regarding model implementation. All references to grid search, hyperparameter tuning and optimisation procedures have been reconciled with the actual modelling process. The revised manuscript consistently states that fixed model parameters were used throughout the analysis and that no additional hyperparameter optimisation was performed.

Comment 3:

Please submit a clean revised manuscript without formatting or tracked-change artifacts. The current file appears to contain residual formatting markers and duplicated or inconsistent section/table numbering.

Response:

This issue has been addressed. A clean revised manuscript has been prepared and submitted. Formatting artefacts, duplicated numbering, and section/table numbering inconsistencies have been corrected. All section headings, table references, figure references and numbering sequences have been reviewed and standardised throughout the manuscript.

Comment 4:

Please continue to frame the study as retrospective spatial intensity modelling rather than real-time forecasting or operational prediction and remove any remaining claims that imply early-warning or deployment-ready predictive use unless they are directly supported by the analysis.

Response:

Thank you for this important observation. The manuscript has undergone a further review to ensure consistent framing of the study as a retrospective spatial intensity modelling exercise based on historical ISIS/ISIL incident data. Additional revisions were made throughout the manuscript to remove or modify terminology that could imply real-time forecasting, operational prediction, early-warning functionality or deployment-ready applications. The revised manuscript now consistently presents the framework as a historical spatial intensity modelling approach intended for exploratory spatial analysis and scenario-based learning.

We sincerely thank the Editor for the valuable guidance provided during the revision process. The additional comments have significantly improved the clarity, consistency and presentation of the manuscript, and we hope that the revised version will now be suitable for further consideration.

Dr. Rubasin Gamage Niluka Lakmali

Corresponding Author

On behalf of all authors

Attachment Submitted filename: Response to Aditional Reviewer 08 June 2026.docx

# Editor report: Decision Letter 2

**DOI:** 10.1371/journal.pone.0355100.r005

**Authors:** Diya Li

6 Jul 2026

PONE-D-26-06707R2

Spatial Intensity Modelling of ISIS-Related Attacks Using Random Forest Regression: A GeoAI-Based Analysis of GTD Data (2012–2019)

PLOS One

Dear Dr. Lakmali,

Thank you for submitting your manuscript to PLOS ONE. After careful consideration, we feel that it has merit but does not fully meet PLOS ONE’s publication criteria as it currently stands. Therefore, we invite you to submit a revised version of the manuscript that addresses the points raised during the review process.

Please submit your revised manuscript by Aug 20 2026 11:59PM. If you will need more time than this to complete your revisions, please reply to this message or contact the journal office at plosone@plos.org. When you're ready to submit your revision, log on to https://www.editorialmanager.com/pone/ and select the 'Submissions Needing Revision' folder to locate your manuscript file.

Please include the following items when submitting your revised manuscript:

- A letter that responds to each point raised by the academic editor and reviewer(s). You should upload this letter as a separate file labeled 'Response to Reviewers'.
- A marked-up copy of your manuscript that highlights changes made to the original version. You should upload this as a separate file labeled 'Revised Manuscript with Track Changes'.
- An unmarked version of your revised paper without tracked changes. You should upload this as a separate file labeled 'Manuscript'.

If you would like to make changes to your financial disclosure, please include your updated statement in your cover letter. Guidelines for resubmitting your figure files are available below the reviewer comments at the end of this letter.

If applicable, we recommend that you deposit your laboratory protocols in protocols.io to enhance the reproducibility of your results. Protocols.io assigns your protocol its own identifier (DOI) so that it can be cited independently in the future. For instructions see: https://journals.plos.org/plosone/s/submission-guidelines#loc-laboratory-protocols. Additionally, PLOS ONE offers an option for publishing peer-reviewed Lab Protocol articles, which describe protocols hosted on protocols.io. Read more information on sharing protocols at https://plos.org/protocols?utm_medium=editorial-email&utm_source=authorletters&utm_campaign=protocols.

As the corresponding author, your ORCID iD is verified in the submission system and will appear in the published article. PLOS supports the use of ORCID, and we encourage all coauthors to register for an ORCID iD and use it as well. Please encourage your coauthors to verify their ORCID iD within the submission system before final acceptance, as unverified ORCID iDs will not appear in the published article. *Only*  the individual author can complete the verification step; PLOS staff *cannot*  verify ORCID iDs on behalf of authors.

We look forward to receiving your revised manuscript.

Kind regards,

Diya Li

Guest Editor

PLOS One

Journal Requirements:

1. If the reviewer comments include a recommendation to cite specific previously published works, please review and evaluate these publications to determine whether they are relevant and should be cited. There is no requirement to cite these works unless the editor has indicated otherwise.

2. Please review your reference list to ensure that it is complete and correct. If you have cited papers that have been retracted, please include the rationale for doing so in the manuscript text, or remove these references and replace them with relevant current references. Any changes to the reference list should be mentioned in the rebuttal letter that accompanies your revised manuscript. If you need to cite a retracted article, indicate the article’s retracted status in the References list and also include a citation and full reference for the retraction notice.

Additional Editor Comments:

Thank you for the revised manuscript and the detailed response. The revision has moved in the right direction, but several issues remain and should be addressed before the manuscript can proceed.

1. Comparative baselines: Section 4.1 now includes a comparative table, but it does not address the specific baselines requested in the previous decision letter. Please add the naive mean baseline, coordinate-only model, and linear regression model, using the same metrics as the Random Forest model (R2, RMSLE, and MAE), or clearly justify any baseline that cannot be implemented. The current comparison is limited to Decision Tree, AdaBoost, XGBoost, and Random Forest.

2. Model tuning: Please remove the remaining inconsistent text stating that hyperparameters were "tuned via grid search" and reconcile all language with the stated use of fixed/default scikit-learn parameters and no additional hyperparameter optimization.

3. Clean manuscript and numbering: Please submit a clean version without tracked-change or formatting artifacts. The current PDF still appears to include residual artifacts such as "Formatted: Font: Not Bold", merged replacement text such as "estimatepredict", and inconsistent numbering such as "4.21" and "Table 32/Table 2".

4. Scope of claims: Please continue to frame the work as retrospective spatial intensity modeling based on historical data. Any remaining language that suggests forecasting, real-time prediction, operational deployment, or prescriptive risk assessment should be removed or clearly limited as future work beyond the current analysis.

Please provide a revised clean manuscript and a point-by-point response identifying where each change was made.

[Note: HTML markup is below. Please do not edit.]

[NOTE: If reviewer comments were submitted as an attachment file, they will be attached to this email and accessible via the submission site. Please log into your account, locate the manuscript record, and check for the action link "View Attachments". If this link does not appear, there are no attachment files.]

To ensure your figures meet our technical requirements, please review our figure guidelines: https://journals.plos.org/plosone/s/figures

You may also use PLOS’s free figure tool, NAAS, to help you prepare publication quality figures: https://journals.plos.org/plosone/s/figures#loc-tools-for-figure-preparation.

NAAS will assess whether your figures meet our technical requirements by comparing each figure against our figure specifications.

# Author comment: Author response to Decision Letter 3

**DOI:** 10.1371/journal.pone.0355100.r006

12 Jul 2026

Response to Reviewers

Manuscript: PONE-D-26-06707R2

Title: Spatial Intensity Modelling of ISIS-Related Attacks Using Random Forest Regression: A GeoAI-Based Analysis of GTD Data (2012–2019)

Dear Dr. Li,

We thank the Guest Editor for the additional careful review of our manuscript and for the constructive comments provided. We have addressed each of the four points raised in the Additional Editor Comments below, along with the two Journal Requirements. All changes were made conservatively, preserving the original structure, methods, results, tables, and conclusions, and are limited to the specific sentences and table entries identified below. A point-by-point response follows.

1. Comparative Baselines

"Section 4.1 now includes a comparative table, but it does not address the specific baselines requested in the previous decision letter. Please add the naive mean baseline, coordinate-only model, and linear regression model, using the same metrics as the Random Forest model (R2, RMSLE, and MAE), or clearly justify any baseline that cannot be implemented."

Response: We have added all three requested baselines to Table 2 (Section 4.1), evaluated using the same three metrics (R², RMSLE, MAE) as the other models in the comparison:

Naive Mean Baseline: R² = 0.00, RMSLE = 0.391, MAE = 0.188

Coordinate-Only Model: R² = 0.41, RMSLE = 0.338, MAE = 0.161

Linear Regression: R² = 0.56, RMSLE = 0.317, MAE = 0.149

These are presented alongside the existing Decision Tree, AdaBoost, XGBoost, and Random Forest results, in order of increasing model complexity. The introductory sentence to Section 4.1 and the closing interpretive paragraph were both updated to describe the full comparison set and the resulting performance progression.

2. Model Tuning

"Please remove the remaining inconsistent text stating that hyperparameters were “tuned via grid search” and reconcile all language with the stated use of fixed/default scikit-learn parameters and no additional hyperparameter optimization."

Response: We confirm that the phrase “tuned via grid search” does not appear anywhere in the current manuscript text. To remove any remaining ambiguity, we revised two passages that referenced cross-validation in a way that could be misread as implying parameter selection:

In Section 4.2, the description of the training procedure now explicitly states that the fixed parameters in Table 1 were used throughout, and that 5-fold cross-validation was used only to assess the stability of model performance, not to select or tune parameters.

In Section 4.6, the description of the robustness checks was revised to explicitly tie the cross-validation and shuffled/stratified partition checks back to the same fixed-parameter configuration.

These edits ensure that every mention of cross-validation in the manuscript is consistent with the fixed-parameter, no-tuning approach described in Section 3.3.1.

3. Clean Manuscript and Numbering

"Please submit a clean version without tracked-change or formatting artifacts. The current PDF still appears to include residual artifacts such as “Formatted: Font: Not Bold”, merged replacement text such as “estimatepredict”, and inconsistent numbering such as “4.21” and “Table 32/Table 2”."

Response: We reviewed the manuscript file directly at the XML level and confirmed that none of the specific artifacts described (residual “Formatted: Font” comments, the merged term “estimatepredict”, section numbering such as “4.21”, or duplicated table numbering such as “Table 32/Table 2”) are present in the current file. Section numbering runs sequentially from 4.1 through 4.7, and all five tables are numbered sequentially with no duplication.

During this review we did identify and correct a small number of leftover punctuation artifacts (isolated duplicate periods) introduced by the editing process itself. These have been removed. All tracked changes have been accepted in the clean “Manuscript” file submitted alongside this letter, and a separate “Revised Manuscript with Track Changes” file is provided showing the full history of edits for this and the previous revision round.

4. Scope of Claims

"Please continue to frame the work as retrospective spatial intensity modeling based on historical data. Any remaining language that suggests forecasting, real-time prediction, operational deployment, or prescriptive risk assessment should be removed or clearly limited as future work beyond the current analysis."

Response: We revised eight passages across Sections 1, 2, 3, 3.1, 3.5, 4.5, and 5.3 that used language implying operational deployment, prescriptive planning, or active forecasting capability. In each case the wording was replaced with language framing the work as retrospective, exploratory spatial intensity analysis. Specific changes include:

Section 1: Removed language suggesting the model output could be used by “planners and law enforcement organizations” to “prioritize resource allocation” and perform “risk-based assessments,” replacing it with a description of the visualizations as a retrospective reference point for future exploratory analysis.

Section 1: Removed the phrase “actionable intelligence to the stakeholders of an urban security plan,” replacing it with language describing the contribution as supporting retrospective, exploratory understanding for researchers and analysts.

Section 2: Revised language describing the framework as informing “security operations” and bridging “decision support and predictive analytics in counter-terrorism planning,” replacing it with a description of the work as a repeatable GeoAI process for retrospective spatial intensity modelling.

Section 3 (Methodology introduction) and Section 3.1: Replaced references to a “prediction model” with “spatial intensity model” and removed the phrase “operationally significant scope,” replacing it with “clearly defined and analytically tractable scope.”

Section 3.5: Revised language describing the visualization workflow as filling “the gap between predictive modelling and practical applicability” and enabling “strategic planning,” replacing it with language describing the visualizations as supporting exploratory interpretation of the modelling results. Similarly revised a passage describing the findings as making the model “more actionable” for “real-life plans associated with counterterrorism and urban security planning,” replacing it with language describing the visualization as supporting exploratory interpretation rather than operational planning use.

Section 4.5: Revised the Figure 3 caption from “The geospatial distribution of predicted threat intensities” to “The geospatial distribution of estimated spatial intensity values,” for consistency with the retrospective framing used elsewhere.

Section 5.3: Revised the passage describing the model as supporting “situational awareness and resource distribution in the high-risk urban setting” and “augmenting proactive security planning and reducing operational cost,” replacing it with language describing the outputs as useful for exploratory, retrospective purposes in resource-constrained research contexts, explicitly noting that any operational use would require further validation.

Sections 5.4 through 5.6 and the Conclusion already framed the work appropriately as retrospective and exploratory in the previous revision and were left unchanged.

Journal Requirements

1. Citations recommended by reviewers: No specific additional citations were recommended by the editor in this decision letter beyond the four numbered comments addressed above; accordingly, no changes were made to the reference list on this basis.

2. Reference list completeness: We reviewed the reference list and confirm that it is complete and correct, and that it does not include any retracted publications.

We thank the Guest Editor again for the guidance provided through this review process and believe the revised manuscript now fully addresses the outstanding concerns. We look forward to the Editor's further consideration.

Attachment Submitted filename: Response to Reviewers pdf 12 July.pdf

# Editor report: Decision Letter 3

**DOI:** 10.1371/journal.pone.0355100.r007

**Authors:** Diya Li

17 Jul 2026

Spatial Intensity Modelling of ISIS-Related Attacks Using Random Forest Regression: A GeoAI-Based Analysis of GTD Data (2012–2019)

PONE-D-26-06707R3

Dear Dr. Lakmali,

We’re pleased to inform you that your manuscript has been judged scientifically suitable for publication and will be formally accepted for publication once it meets all outstanding technical requirements.

Within one week, you’ll receive an e-mail detailing the required amendments. When these have been addressed, you’ll receive a formal acceptance letter and your manuscript will be scheduled for publication.

An invoice will be generated when your article is formally accepted. Please note, if your institution has a publishing partnership with PLOS and your article meets the relevant criteria, all or part of your publication costs will be covered. Please make sure your user information is up-to-date by logging into Editorial Manager at Editorial Manager® and clicking the ‘Update My Information' link at the top of the page. For questions related to billing, please contact billing support.

If your institution or institutions have a press office, please notify them about your upcoming paper to help maximize its impact. If they’ll be preparing press materials, please inform our press team as soon as possible -- no later than 48 hours after receiving the formal acceptance. Your manuscript will remain under strict press embargo until 2 pm Eastern Time on the date of publication. For more information, please contact onepress@plos.org.

Kind regards,

Diya Li

Guest Editor

PLOS One

Additional Editor Comments (optional):

Reviewers' comments:

# Editor report: Acceptance letter

**DOI:** 10.1371/journal.pone.0355100.r008

**Authors:** Diya Li

PONE-D-26-06707R3

PLOS One

Dear Dr. Lakmali,

I'm pleased to inform you that your manuscript has been deemed suitable for publication in PLOS One. Congratulations! Your manuscript is now being handed over to our production team.

At this stage, our production department will prepare your paper for publication. This includes ensuring the following:

* All references, tables, and figures are properly cited

* All relevant supporting information is included in the manuscript submission,

* There are no issues that prevent the paper from being properly typeset

You will receive further instructions from the production team, including instructions on how to review your proof when it is ready. Please keep in mind that we are working through a large volume of accepted articles, so please give us a few days to review your paper and let you know the next and final steps.

Lastly, if your institution or institutions have a press office, please let them know about your upcoming paper now to help maximize its impact. If they'll be preparing press materials, please inform our press team within the next 48 hours. Your manuscript will remain under strict press embargo until 2 pm Eastern Time on the date of publication. For more information, please contact onepress@plos.org.

You will receive an invoice from PLOS for your publication fee after your manuscript has reached the completed accept phase. If you receive an email requesting payment before acceptance or for any other service, this may be a phishing scheme. Learn how to identify phishing emails and protect your accounts at https://explore.plos.org/phishing.

If we can help with anything else, please email us at customercare@plos.org.

Thank you for submitting your work to PLOS ONE and supporting open access.

Kind regards,

PLOS ONE Editorial Office Staff

on behalf of

Dr. Diya Li

Guest Editor

PLOS One
